# CCL5/CCR5-mediated peripheral inflammation exacerbates blood‒brain barrier disruption after intracerebral hemorrhage in mice

**DOI:** 10.1186/s12967-023-04044-3

**Published:** 2023-03-14

**Authors:** Jie Lin, Ya Xu, Peiwen Guo, Yù-Jié Chen, Jiru Zhou, Min Xia, Binbin Tan, Xin Liu, Hua Feng, Yujie Chen

**Affiliations:** 1 Department of Neurosurgery and State Key Laboratory of Trauma, Burn and Combined Injury, Southwest Hospital, Third Military Medical University (Army Medical University), 29 Gaotanyan Street, Shapingba District, Chongqing, 400038 China; 2 Chongqing Clinical Research Center for Neurosurgery, Southwest Hospital, Third Military Medical University (Army Medical University), Chongqing, 400038 China; 3 Chongqing Key Laboratory of Precision Neuromedicine and Neuroregenaration, Southwest Hospital, Third Military Medical University (Army Medical University), Chongqing, 400038 China; 4 Department of Neurosurgery, The First Affiliated Hospital of Chongqing Medical University, Chongqing, 400016 China; 5 Clinical Medical Research Center, Southwest Hospital, Third Military Medical University (Army Medical University), Chongqing, 400038 China

**Keywords:** Intracerebral hemorrhage, Blood‒brain barrier, C–C chemokine receptor 5, Peripheral inflammation, Neuroinflammation, Brain-periphery crosstalk

## Abstract

**Background:**

Owing to metabolic disequilibrium and immune suppression, intracerebral hemorrhage (ICH) patients are prone to infections; according to a recent global analysis of stroke cases, approximately 10 million new-onset ICH patients had experienced concurrent infection. However, the intrinsic mechanisms underlying the effects of infection related peripheral inflammation after ICH remain unclear.

**Methods:**

Lipopolysaccharide (LPS) was intraperitoneally injected into ICH model mice to induce peripheral inflammation. Neurobehavioral deficits, blood‒brain barrier (BBB) disruption, and the expression of CCR5, JAK2, STAT3, and MMP9 were evaluated after treatment with recombinant CCL5 (rCCL5) (a CCR5 ligand), maraviroc (MVC) (an FDA-approved selective CCR5 antagonist), or JAK2 CRISPR plasmids.

**Results:**

Our study revealed that severe peripheral inflammation increased CCL5/CCR5 axis activation in multiple inflammatory cell types, including microglia, astrocytes, and monocytes, and aggravated BBB disruption and neurobehavioral dysfunction after ICH, possibly in part through the JAK2/STAT3 signaling pathway.

**Conclusions:**

CCR5 might be a potential target for the clinical treatment of infection-induced exacerbation of BBB disruption following ICH.

**Supplementary Information:**

The online version contains supplementary material available at 10.1186/s12967-023-04044-3.

## Background

Intracerebral hemorrhage (ICH), one of the most common types of stroke, mainly occurs in the basal ganglia and has a mortality of up to 38.67% [1]. Surviving patients often experience multiple irreversible functional injuries and sequelae after discharge [2]. After ICH onset, secondary injury is generally caused via inflammation following the destruction of brain structures, resulting in subsequent damage to the blood‒brain barrier (BBB) and aggravation of cerebral edema [3–5]. Owing to metabolic disequilibrium and immune suppression, ICH patients are prone to infections, including pulmonary and intestinal infections [6, 7]. According to a global analysis of stroke cases, approximately 10 million new-onset ICH patients had experienced concurrent infection [1], and in our previous study, almost one-third of ICH patients in the neurosurgical intensive care unit developed secondary sepsis [8]. Sepsis exacerbates the above-mentioned inflammatory damage after ICH since terminal cytokine receptors on the vagal nerve can detect visceral inflammation and feedback to furtherly stimulate cholinergic nuclei and innate immune cells in the brain [9]. Other mechanisms, also contribute to the crosstalk between central nervous system inflammation and peripheral inflammation, including aberrant infiltration of additional peripheral monocytes [10]. However, the intrinsic mechanisms underlying the effects of severe peripheral inflammation in ICH still remain unclear [11].

C–C chemokine receptor 5 (CCR5) is a chemokine receptor mainly composed of a 7-transmembrane-α-helix and an intracellular C-terminus [12]. It can be activated via a variety of cytokines, including C–C chemokine ligand 5 (CCL5), and is widely expressed on various cell types [13], e.g., microglia, astrocytes, monocytes, and neurons. Previous studies have found that CCR5 is involved in the proliferation and activation of immunocytes as well as the production of cytokines. In particular, it is thought to play a key role in immunocyte trafficking under physiological and pathological conditions [14]. During infection, increased production of CCR5 ligands can lead to CCR5 overexpression and the recruitment of CCR5^+^ cells to areas around lesions, resulting in amplification of the local inflammatory response [14].

Previous research has confirmed that CCL5-CCR5 signaling pathway activation could significantly promote neuroinflammation and neurological deficits [12]. However, whether CCR5 signaling participates in central nervous system impairment after ICH with comorbid severe peripheral inflammation has not been exactly explored [11]. In addition, Janus kinase 2 (JAK2), a nonreceptor protein tyrosine kinase, has been reported to mediate the transduction of CCR5 signals from the extracellular space to the intracellular space by phosphorylating the tyrosine residues of various target proteins to activate their intracellular domains [15]. Signal transducer and activator of transcription 3 (STAT3) is downstream of JAK2 and has been confirmed to modulate the secretion of multiple proteolytic enzymes, including matrix metalloproteinase-9 (MMP9) [15–17]. The JAK2/STAT3/MMP9 signaling pathway is a classical inflammatory pathway that has been reported to be involved in tight junction protein [e.g., zonula occludens-1 (ZO-1) and claudin-5 (CLDN5)] elimination and neuroinflammatory injury [18]. Thus, we hypothesized in the current study that upregulation of CCL5/CCR5 expression exacerbates BBB disruption, which further aggravates cerebral edema and neurobehavioral dysfunction in mice with severe peripheral inflammation after ICH, possibly via JAK2/STAT3-mediated BBB disruption.

## Methods

### Animals

All experimental procedures performed in this study were reviewed and approved by the Laboratory Animal Welfare and Ethics Committee of Third Military Medical University (AMUWEC2020762). All protocols were performed in according with the National Institutes of Health Guide for the Care and Use of Laboratory Animals and are reported following the Animal Research: Reporting of In Vivo Experiments, Version 2.0 (ARRIVE 2.0) guidelines. A total of 326 8- to 12-week-old male C57BL/6N mice weighing 24–28 g were provided by the Animal Experimental Center of Third Military Medical University (Chongqing, China). The animals housed at an optimal temperature and humidity on a regular 12 h day/night cycle. They were randomly divided into different experimental groups and provided ad libitum access to food and water.

### Experimental design

**Fig. 1:**
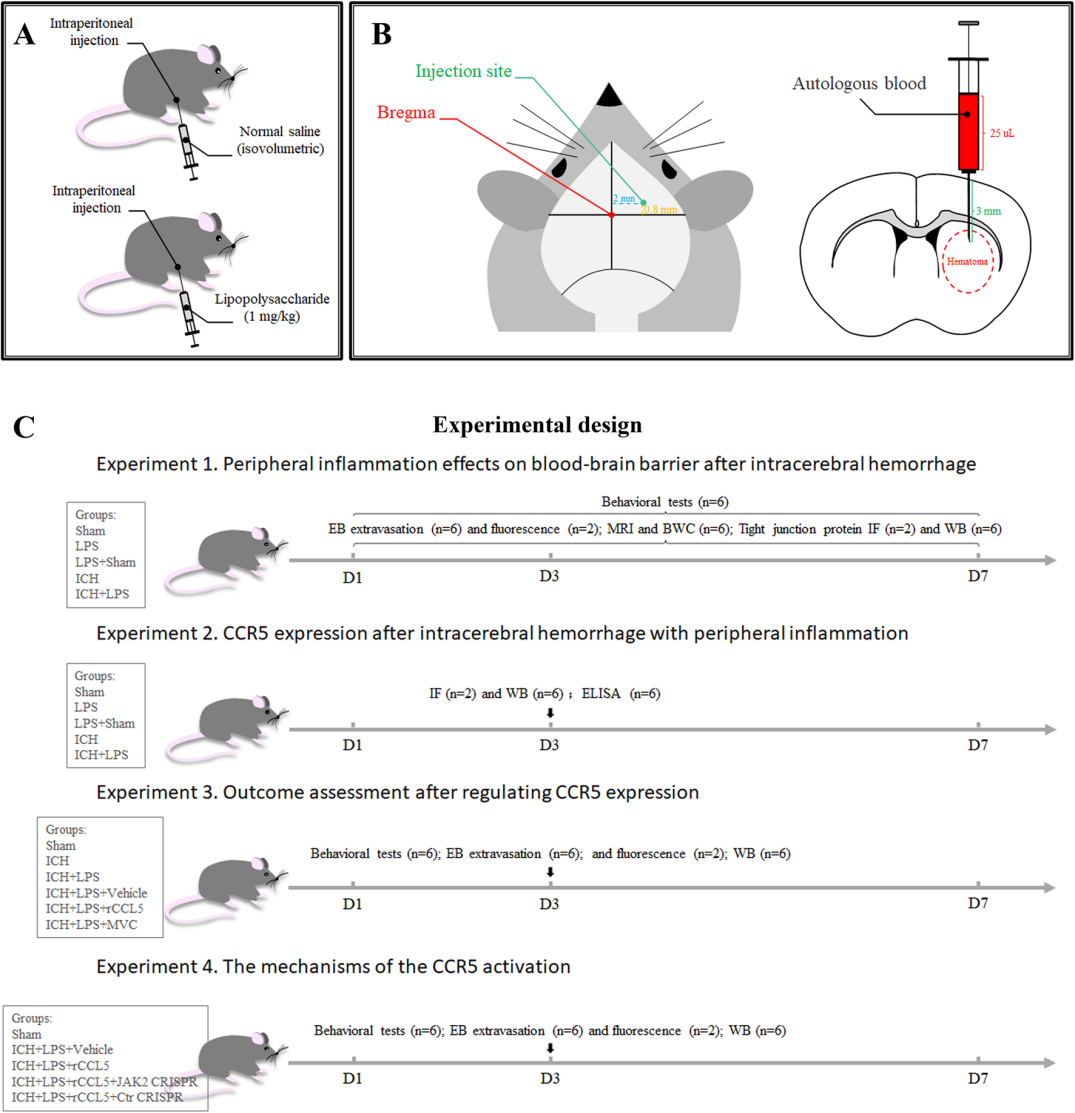
Animal model construction and experimental design. **A** I.p. drug administration in the experimental and control groups. **B** Localization and volume of the hematoma in the ICH model. **C** Experimental design. LPS: lipopolysaccharide; EB: Evans blue; MRI: magnetic resonance imaging; BWC: brain water content; IF: immunofluorescence; WB: Western blot; ELISA: enzyme-linked immunosorbent assay; rCCL5: recombinant chemokine ligand 5; MVC: maraviroc; Ctr: control

This study consisted of four parts, and the relevant timelines are displayed in Fig. 1. The number of mice required for each experimental group and the mortality rate for each group were calculated as illustrated in Additional file 1: Table S1. All experiments and data analysis were performed by researchers blinded to the group information.

*Experiment 1* The effects of peripheral inflammation on the BBB and neurobehavioral function after ICH were assessed via behavioral tests (n = 6), the Evans Blue (EB) extravasation assay (n = 6), Evans blue fluorescence analysis (n = 2), brain water content (n = 6), and magnetic resonance imaging (MRI) (n = 1). A total of 189 mice were randomly divided into five groups: the sham, lipopolysaccharide (LPS), LPS + sham, ICH, and ICH + LPS groups. The mice in all groups except the sham group were analyzed at three timepoints, i.e., 1 day, 3 days, and 7 days post-ICH. The number of mice used for each assay at each time point is described above. An additional 8 mice from each group were used for double immunofluorescence staining (n = 2) and western blotting (n = 6) 3 days after ICH to evaluate the expression of tight junction proteins, including ZO-1 and CLDN5.

*Experiment 2* The animals were grouped as described for Experiment 1 and were only analyzed at 3 days after ICH. The expression of CCR5, p-JAK2, p-STAT3, and MMP9 in right hemisphere tissues from mice in each group (n = 6) was measured by Western blotting. In addition, an enzyme-linked immunosorbent assay (ELISA) was conducted to evaluate the CCL5 level in both right hemisphere tissues and serum samples from each group (n = 6). The cellular localization of CCR5 in each group was assessed via double immunostaining (n = 2) for glial fibrillary acidic protein (GFAP, an astrocyte marker), myeloperoxidase (MPO, a monocyte marker), and ionized calcium binding adapter molecule-1 (IBA-1, a microglial marker).

*Experiment 3* To assess the effects of changes in CCR5 expression, mice were randomly divided into six groups: the sham, ICH, ICH + LPS, ICH + LPS + vehicle, ICH + LPS + recombinant CCL5 (rCCL5), and ICH + LPS + maraviroc (MVC) groups. BBB integrity and neurobehavioral function in each group were evaluated 3 days after ICH via behavioral tests (n = 6), the Evans Blue (EB) extravasation assay (n = 6) and Evans blue fluorescence analysis (n = 2). Then, western blotting (n = 6) was performed to examine the expression of p-JAK2, p-STAT3, and MMP9 in each group 3 days post-ICH.

*Experiment 4* To further determine the detrimental effects of CCR5/JAK2 activation, mice were given intracerebral ventricle (i.c.v.) injection of a JAK2 CRISPR knockout plasmid or control CRISPR plasmid before ICH modeling. An additional 16 mice were randomly divided into two groups: the ICH + LPS + rCCL5 + JAK2 CRISPR (n = 8) and ICH + LPS + rCCL5 + Ctr CRISPR (n = 8) groups. Behavioral tests and the EB extravasation assay were performed 3 days after ICH.

### Animal models

#### ICH model

ICH model construction was performed as our previously described by our group [19]. Briefly, the mice were anesthetized with isoflurane (RWD Life Sciences Ltd., China). After the skin on the head was prepared, the mice were fixed to a stereotaxic instrument (RWD Life Sciences Ltd., China) in the prone position. Bregma was used as a reference to locate the appropriate brain region Then, the internal capsule was located and labeled with the stereotaxic instrument (coordinates: 0.8 mm anterior to bregma and 2.0 mm lateral to the midline). A small burr hole (diameter of approximately 1 mm) was made in the skull with a high-speed cranial drill. A sterile Hamilton syringe was to inject 25 μl autologous blood into the right basal ganglia (3 mm deep from the dura) at a rate of 2 μl/min. After injection, the needle was kept in place for an additional 5 min and then removed slowly. In the sham group, the mice were anesthetized and fixed to the stereotaxic instrument, the skin was disinfected, the internal capsule was located and a hole was made in the skull as described above. After the syringe was removed, bone wax was used to seal the hole in the skull, the surgical site was disinfected and sutured, and the mice were placed on a heating pad until they woke up. The operation was performed under strict aseptic conditions on an ultraclean table sterilized by ultraviolet radiation.

#### Model of ICH with sustained peripheral inflammation

The concentration of lipopolysaccharide (LPS) was selected according to previous reports [20, 21]. ELISA kits (ab222503 and ab208348; Abcam) were used to verify that the levels of inflammatory factors, including interleukin-6 (IL-6) and tumor necrosis factor-α, in the serum were elevated to confirm that peripheral inflammation was induced by intraperitoneal (i.p.) injection of LPS (L2880; Sigma‒Aldrich) (Additional file 1: Fig. S1). ICH was modeled as described above. LPS was dissolved in sterile normal saline (NS) and administered to mice at a dose of 1 mg/kg. LPS was administered 24 h before, 1 h after and 24 h after ICH. Mice in the sham group and ICH group were injected with an equal volume of NS at the same time point.

#### Drug administration

MVC, an FDA-approved selective CCR5 antagonist [14], was purchased from Selleck Chemicals and dissolved in 1% dimethyl sulfoxide (DMSO). The mice were administered 100 mg/kg MVC via i.p. injection 25 h before, 2 h after and 23 h after ICH [22]. rCCL5 (0.5 μg/mouse; 478-MR-025; R&D Systems) was dissolved in the same vehicle and intraperitoneally injected into mice 25 h before, 2 h after and 23 h after ICH [12]. To specifically knockdown JAK2 expression, mice were administered a JAK2 CRISPR knockout plasmid (2 μl/mouse; sc-421200; Santa Cruz Biotechnology, Shanghai, China) or control CRISPR plasmid (2 μl/mouse; sc-418922; Santa Cruz Biotechnology, Shanghai, China) via intracerebroventricular (i.c.v.) injection 25 h before ICH induction [23].

#### Intraventricular injection

Intraventricular injection was performed as reported previously [24]. The mice were deeply anesthetized with isoflurane (R510-22; RWD Life Sciences Ltd., China), and a burr hole was made in the skull at particular coordinates (0.22 mm posterior to bregma and 1.0 mm lateral to the midline) with a stereotaxic instrument (RWD Life Sciences Ltd., China). The CRISPR plasmids were delivered into the right lateral ventricle at a depth of 2.25 mm from the dura with a 5 μl Hamilton syringe at a rate of 0.667 μl/min through the burr hole. After the syringe was removed slowly, the hole was sealed with bone wax.

### Behavioral tests

#### Open field test (OFT)

The OFT was used to measure the exploratory behavior of the mice [25]. The OFT box was divided equally into four squares (each square: 50 cm × 50 cm × 40 cm). Before the experiment, the mice were habituated to the box for 1 h, and the appropriate parameters were selected in Viewpoint software (Consent Biotechnology Co. LTD, Changsha, China). Then, the mice were individually placed in the center of each square at the same position and in the same direction and recorded for 5 min. The trajectories of the mice and the distance traveled were recorded by a video imaging system (Consent Biotechnology Co. LTD, Changsha, China), and the average speed was calculated.

#### Basso mouse scale (BMS)

Locomotor coordination was evaluated with the BMS. Scores ranged from 0 (no ankle movement) to 9 (frequent or consistent plantar stepping that was mostly coordinated, parallel orientation of the paws during the initial contact and lifting of the paws, normal trunk stability, and a constantly raised tail) [26].

### MRI and brain water content (BWC) analysis

Brain edema was initially evaluated via a 7.0 T small animal magnetic resonance scanner as previously reported [27]. After the mice were anesthetized with isoflurane (R510-22, RWD Life Sciences Ltd., China), T2 scans of the brain with a 25 mm × 25 mm field and 0.5 mm thickness were taken on days 1, 3, and 7 after ICH induction. Then, brain edema in ipsilateral hemisphere was quantitatively assessed by measuring the BWC of different regions including basal ganglia, cortex, cerebellum, hippocampus, and brainstem (Fig. 3B), via the wet/dry method on days 1, 3, and 7 post-ICH as described in a previous report [28].

### EB extravasation and fluorescence

EB extravasation and fluorescence were measured as reported previously [29, 30]. After disinfection with iodophor, the mice were injected with 2% EB dye (5 ml/kg; E2129; Sigma‒Aldrich) through the tail vein. The EB dye was allowed to circulate in the mice for 2 h before sacrifice. Under continuous anesthesia with isoflurane (R510-22, RWD Life Sciences Ltd. China), the thorax was opened, and the heart was perfused with NS. The brain was immediately harvested, and the right hemisphere was isolated. Then, the tissue was weighed, and NS (400 μl) was added for homogenization. The samples were centrifuged at 15,000×*g* for 30 min. Next, an equal amount of 50% trichloroacetic acid was added to the supernatant. The samples were incubated overnight at 4 °C and centrifuged at 15,000×*g* for 30 min again. The content of EB in the supernatant was then quantified with a spectrophotometer (615 nm; Varioskanfla, Thermo Fisher Scientific) and a standard curve. To analyze EB fluorescence, the brain was harvested promptly after cardiac perfusion with NS and 4% paraformaldehyde, and frozen coronal brain sections (20 μm) were prepared and stored at -20 °C until use. After the sections were stained with 4’-6-diamidino-2-phenylindole (DAPI), EB fluorescence was observed using a confocal laser scanning microscope (mRFP 555; ZISS 880, Carl Zeiss Microscopy Ltd., England).

### Immunofluorescence staining

Immunofluorescence staining of frozen brain sections was performed as described in previous reports [19, 29]. Brain tissues were isolated from the mice 3 days post-ICH induction after transcardial perfusion with NS and 4% paraformaldehyde during deep anesthetization. Then, the brains were immediately fixed in 4% paraformaldehyde for 1 day and dehydrated in 30% sucrose for 3 days. Serial frozen coronal sections (20 μm) were obtained with a freezing microtome (CM1860UV, Leica, Wetzlar, Germany) and permeabilized with 0.5% Triton X-100 in phosphate-buffered saline for 30 min. The sections were sequentially incubated with primary antibodies overnight at 4 °C and fluorescent secondary antibodies for 2 h at room temperature after blocking with 5% bovine serum albumin for 1.5 h at room temperature. The primary antibodies included FITC-conjugated rat monoclonal anti-CCR5 (1:200; ab11466; Abcam), rabbit monoclonal anti-ZO1 (1:200; ab221547; Abcam), rabbit polyclonal anti-CLDN5 (1:200; 34-1600; Thermo Fisher Scientific), rabbit monoclonal anti-MPO (1:250; MA5-42,652; Thermo Fisher Scientific), rabbit monoclonal anti-GFAP (1:200; ab220820; Abcam), rabbit monoclonal anti-IBA1 (1:200; ab178846; Abcam), mouse monoclonal anti-CD31 (1:200; MA1-26,196; Thermo Fisher Scientific), and rabbit polyclonal anti-CD31 (1:200; PA5-32,321; Thermo Fisher Scientific). The secondary antibodies included Alexa Fluor 488- and Alexa Fluor 555-conjugated secondary antibodies against mouse and rabbit (1:500; Abcam). The nuclei were counterstained with DAPI (Santa Cruz Biotechnology). Four fields of view around the perihematomal area in each slice (3 slices/mouse) were observed and analyzed.

### Western blot analysis

Western blotting was performed as described in previous reports [31]. The ipsilateral brain hemisphere was removed and homogenized to obtain total protein with a protein extraction kit (BC3710; Solarbio; Beijing, China) at a low temperature. After electrophoresis, the proteins were transferred from the gel to PVDF membranes, and the PVDF membranes were blocked in 5% bovine serum albumin for 2 h at room temperature and incubated with the following primary antibodies at 4 °C overnight: rabbit polyclonal anti-CCR5 (1:1000; ab65850; Abcam), rabbit polyclonal anti-ZO1 (1:1000; ab216880; Abcam), rabbit polyclonal anti-CLDN5 (1:1000; 34–1600; Thermo Fisher Scientific), rabbit monoclonal anti-STAT3 (1:1000; ab68153; Abcam), rabbit monoclonal anti-pSTAT3 (1:1000; ab267373; Abcam), rabbit monoclonal anti-JAK2 (1:1000; ab108596; Abcam), rabbit monoclonal anti-pJAK2 (1:1000; ab32101; Abcam), rabbit polyclonal anti-MMP9 (1:1000; ab283575; Abcam), and mouse monoclonal anti-glyceraldehyde-3-phosphate dehydrogenase (GAPDH) (1:5000; ab8245; Abcam). GAPDH was used as an internal loading control. After probing with specific horseradish peroxidase-conjugated secondary antibodies (1:10,000; Abcam) for 2 h at room temperature, the immunoreactive bands were visualized and detected with an enhanced chemiluminescence reagent kit (Thermo Scientific, Rockford, IL, USA) and a bioimaging system (ChemiDoc XRS + ; Bio-Rad, Hercules, CA, USA). Image Lab software (Image Lab 3.0; Bio-Rad, Hercules, CA, USA) was used to quantify the gray value of each band.

### ELISA

A CCL5 mouse ELISA kit (ab100739; Abcam) was used to measure the level of CCL5 in the ipsilateral cerebral hemisphere and serum. The brain tissue supernatants, but not the serum samples, were diluted 50 times before the assay. All procedures were performed in strict accordance with the manufacturer’s instructions.

### Statistical analysis

All statistical data were analyzed and diagramed using GraphPad Prism 7.04 (GraphPad Software, USA). Continuous data are expressed as the mean ± SD, while discrete data are presented as the median ± IQR. The two independent variances were compared with student’s T test or Mann–Whitney test. For comparisons among more than two groups, one-way analysis of variance (ANOVA) followed by a post hoc test was used to analyze normally distributed data, and a nonparametric test followed by the Kruskal‒Wallis test was used to analyze nonnormally distributed data. Two-way repeated-measures ANOVA was used to compare behavioral data between different time points and groups. *P* < 0.05 was regarded as statistically significant. And all detail about statistical processes were displayed in Additional file 1.

## Results

### Mortality and exclusion

The mortality rate of the mice was 4.03% (11/273) in this study, and there was no significant difference in mortality rate among the experimental groups. None of the mice were excluded (Additional file 1: Table S1).

### Severe peripheral inflammation exacerbated apoplectic dyskinesia after ICH

**Fig. 2:**
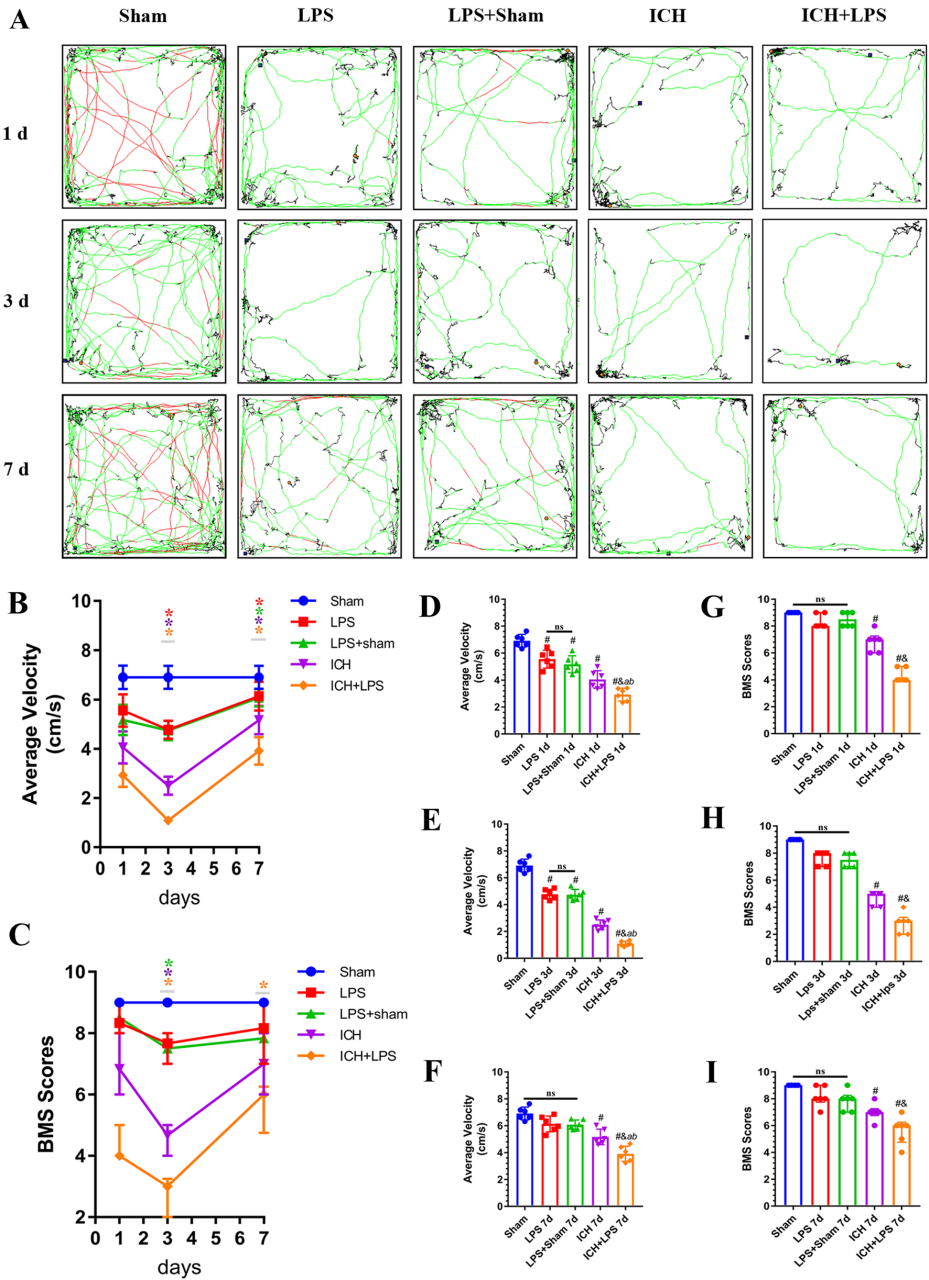
Severe peripheral inflammation exacerbated apoplectic dyskinesia at 1, 3, and 7 days after ICH. **A** Trajectory in the OFT and **B**, **D**-**F** statistical analysis of the average velocity of the mice. According to preset parameters, mouse trajectories are presented in three colors: black (low speed), green (middle speed), and red (high speed). **C**, **G**-**I** Statistical analysis of the BMS scores of the mice. The data are expressed as the mean ± SD or median and range. ^*^ vs. the ICH group at 1 day, *P* < 0.05; ^#^ vs. the sham group, *P* < 0.05; ^*a*^ vs. the LPS group, *P* < 0.05; ^*b*^ vs. the LPS + sham group, *P* < 0.05; ^*&*^ vs. the ICH group, *P* < 0.05; ns: no significance

The motor function of the mice was assessed via the BMS and the OFT on days 1, 3, and 7 after ICH. Decreases in functional scores and average velocity were observed in the experimental groups compared to the sham group, especially at 3 days after ICH (Fig. 2B, C). Moreover, analysis of average velocity and BMS scores revealed that the motor function of ICH + LPS group mice was much worse than that of ICH group mice at 1 day, 3 days and 7 days after ICH (*P* < 0.05; Fig. 2D–I).

### Severe peripheral inflammation aggravated cerebral edema and BBB disruption after ICH

**Fig. 3:**
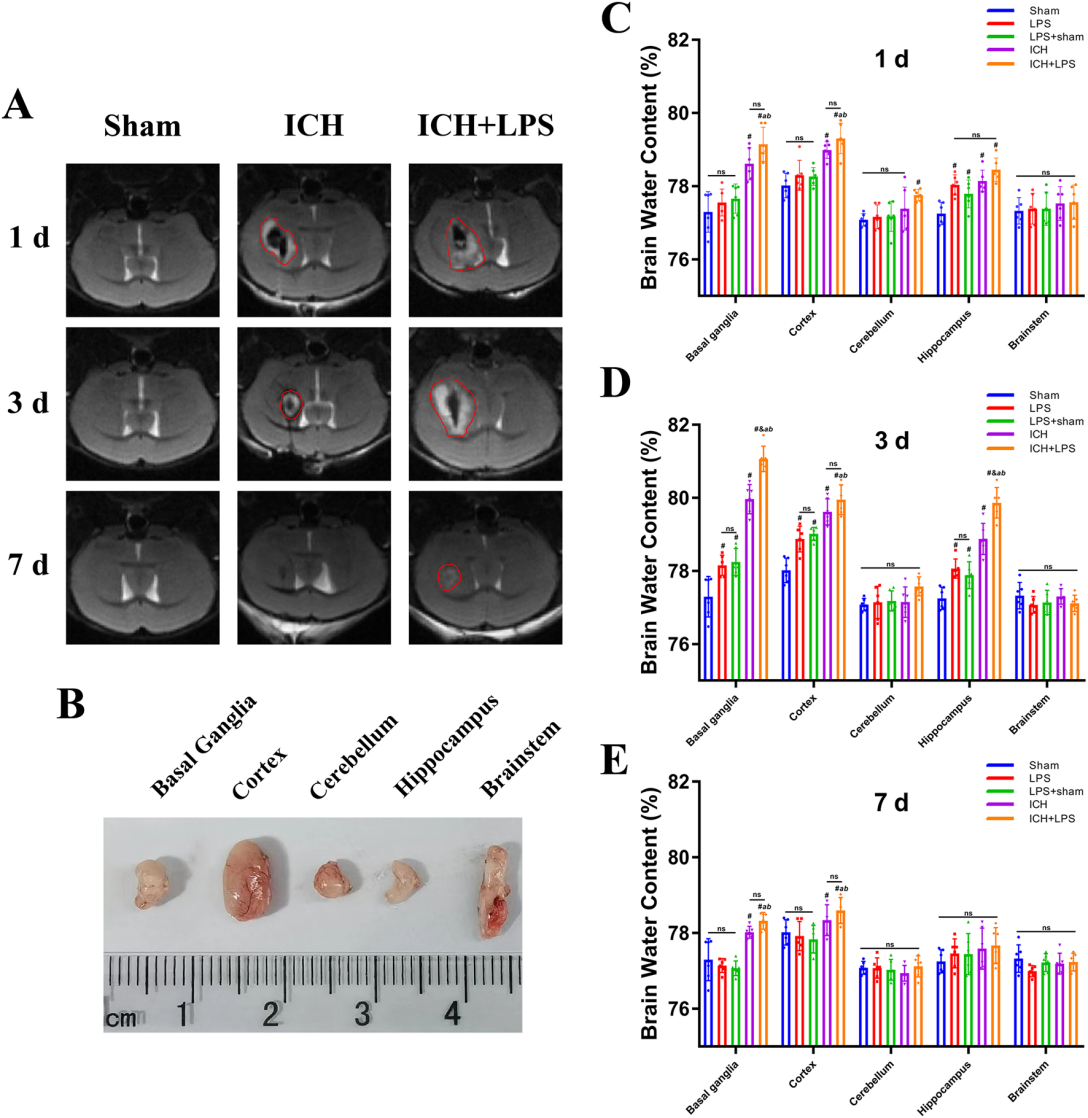
Severe peripheral inflammation aggravated cerebral edema on days 1, 3, and 7 after ICH. **A** Coronal T2 magnetic resonance images. **B** Schematic image of different brain regions in ipsilateral hemisphere, including basal ganglia, cortex, cerebellum, hippocampus, and brainstem. **C**, **D**, **E** Statistical analysis of BWC in different brain regions at 1d, 3d, 7d after ICH. The data are expressed as the mean ± SD. ^#^ vs. the sham group, *P* < 0.05; ^*a*^ vs. the LPS group, *P* < 0.05; ^*b*^ vs. the LPS + sham group, *P* < 0.05; ^*&*^ vs. the ICH group, *P* < 0.05; ns: no significance

**Fig. 4:**
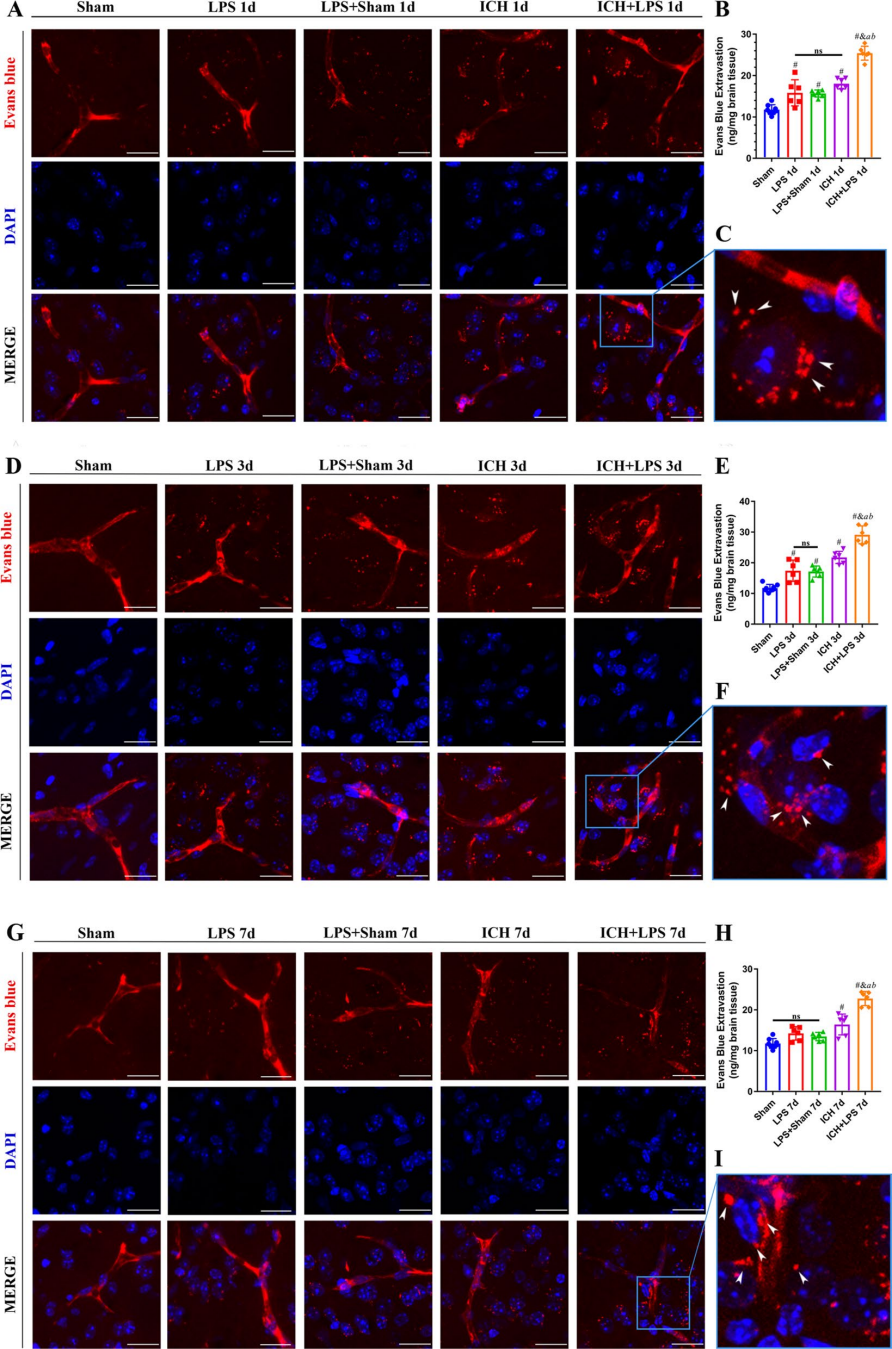
Severe peripheral inflammation exacerbated BBB disruption 1, 3, and 7 days after ICH. **A**, **D**, **G** EB fluorescence images; scale bar = 50 μm. **C**, **F**, **I** Magnified images. The arrows in the magnified images indicate EB dye extravasated from vessels. **B**, **E**, **H** Quantitative analysis of EB extravasation. The data are expressed as the mean ± SD. ^#^ vs. the sham group, *P* < 0.05; ^*a*^ vs. the LPS group, *P* < 0.05; ^*b*^ vs. the LPS + sham group, *P* < 0.05; ^*&*^ vs. the ICH group, *P* < 0.05; ns: no significance

Brain edema was evaluated by MRI and BWC analysis of different encephalic regions at 1, 3, and 7 days after ICH. Nonquantitative analysis of the MRI results indicated that the edema area was wider and the absorption rate was slower in the ICH + LPS group than in the ICH group (Fig. 3A). A significant increase in BWC was observed in the basal ganglia and cortex of ICH + LPS group compared to that of the ICH group at 1 day, 3 days and 7 days after ICH (*P* < 0.05; Fig. 3B-E). Also, the hippocampus regions were inclined to display obvious edema in experimental groups at 3 days after ICH (*P* < 0.05; Fig. 3D). Conversely, cerebellums and brainstems seemed to be unaffected in our results (*P* > 0.05; Fig. 3C–E). We furtherly assessed BBB integrity via the EB extravasation assay. EB extravasation in the ipsilateral (right) hemisphere was significantly increased in the ICH + LPS group compared with the ICH group at 3 days and 7 days after ICH (*P* < 0.05; Fig. 4E, H). In addition, compared with the sham group, the LPS group exhibited a certain increase in EB extravasation in the ipsilateral (right) hemisphere at 1 day and 3 days after ICH (*P* < 0.05; Fig. 4B, E); meanwhile, BWC analysis also reveal obvious cerebral edema especially on the basal ganglia, cortex, and hippocampus areas after 3 days ICH (Fig. 3B–E). The above results provide evidence that cerebral edema and BBB disruption were most severe at 3 days after ICH. Thus, this time point was selected as the main time point for our subsequent experiments.

**Fig. 5:**
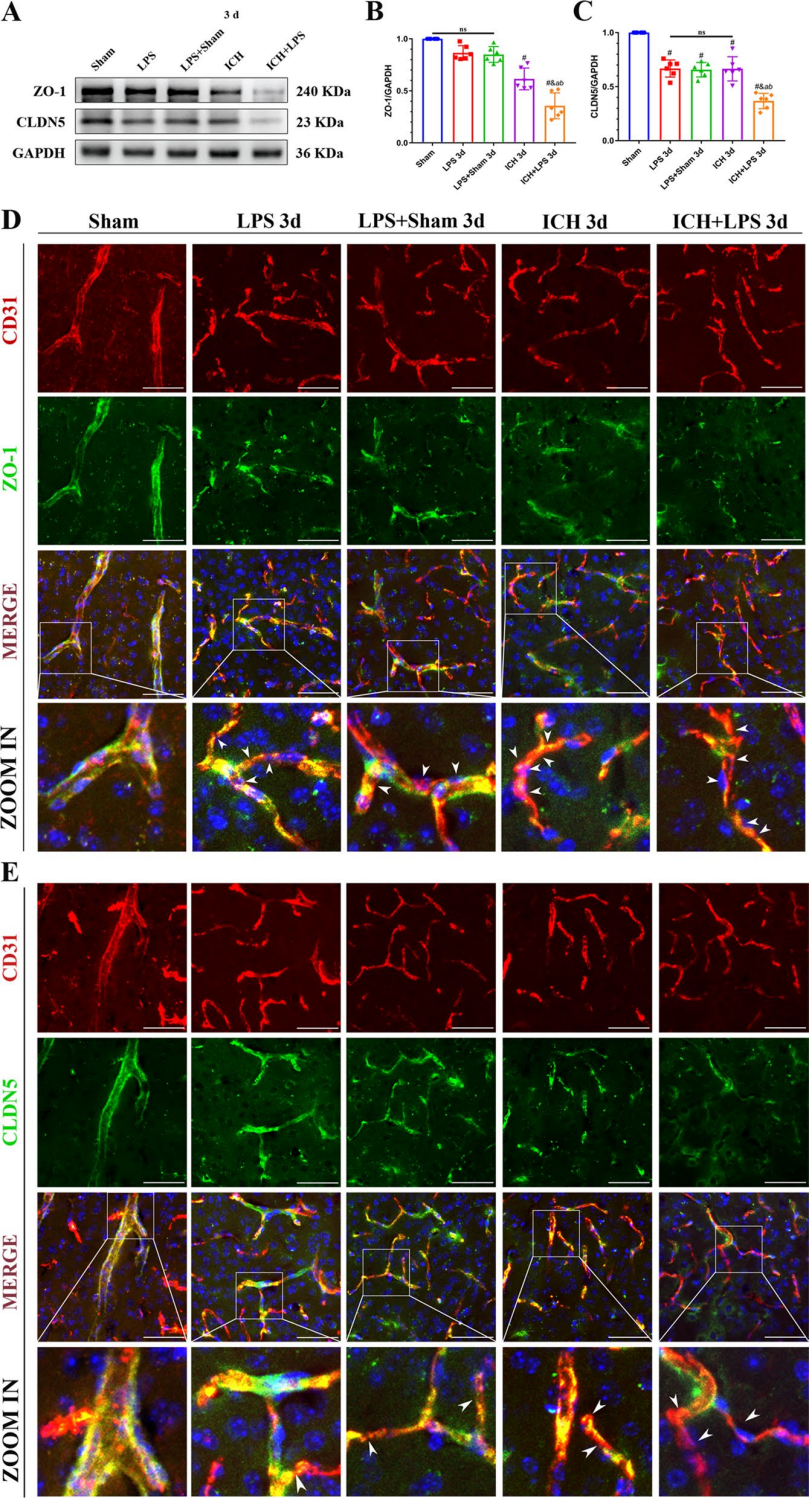
Severe peripheral inflammation downregulated tight junction protein expression 3 days after ICH. **A**–**C** Western blot images and quantitative analysis of ZO-1 and CLDN5 expression. The data are expressed as the mean ± SD. ^#^ vs. the sham group, *P* < 0.05; ^*a*^ vs. the LPS group, *P* < 0.05; ^*b*^ vs. the LPS + sham group, *P* < 0.05; ^*&*^ vs. the ICH group, *P* < 0.05; ns: no significance. **D**, **E** Double immunofluorescence staining images and magnified representative pictures of ZO-1 (green) and CLDN5 (green) in endothelial cells (CD31, red). Scale bar = 50 μm. The arrows in the magnified images indicate regions in which tight junction protein levels are significantly decreased in vessels

At 3 days post-ICH, peripheral inflammation notably decreased the expression of tight junction proteins in the ipsilateral (right) cerebral hemisphere after ICH (Fig. 5). Western blot analysis demonstrated that ZO-1 and CLDN5 expression in the ipsilateral (right) cerebral hemisphere was significantly attenuated in the ICH + LPS group compared to the ICH group (*P* < 0.05; Fig. 5A–C). Double immunostaining for CD31 (an endothelial cell marker) and ZO-1 or CLDN5 further proved these results (Fig. 5D, E).

### Endogenous expression of CCR5 and the CCR5 ligand CCL5 in the brain and periphery after ICH and LPS administration

**Fig. 6:**
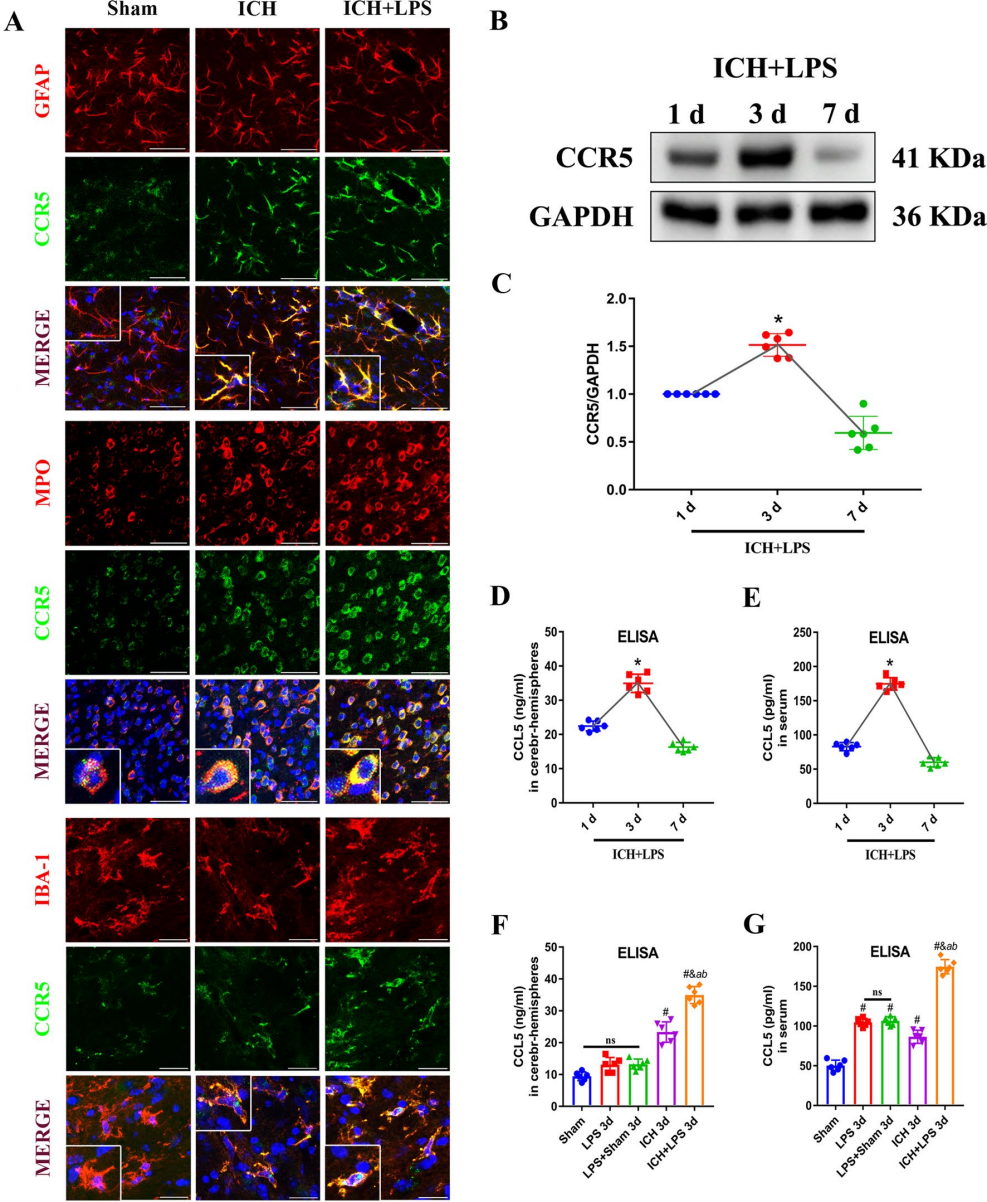
Expression of CCR5 and CCL5/Rantes after ICH and induction of severe peripheral inflammation. **A** Double immunofluorescence staining images and magnified representative images of the localization of CCR5 (green) in astrocytes (GFAP, red, scale bar = 50 μm), monocytes (MPO, red, scale bar = 50 μm), and microglial cells (IBA-1, red, scale bar = 20 μm). **B**, **C** Western blot images and quantitative analysis of the change in CCR5 levels over time in the ipsilateral hemisphere in the ICH + LPS group. **D**, **E** Quantitative analysis of the change in CCL5 levels over time in the ipsilateral hemisphere and serum in the ICH + LPS group. **F**, **G** Quantitative analysis of CCL5 levels in the ipsilateral hemisphere and serum in the sham, LPS, LPS + sham, ICH, and ICH + LPS groups 3 days after ICH. The data are expressed as the mean ± SD. ^*^ vs. the ICH group at 1 day, *P* < 0.05; ^#^ vs. the sham group, *P* < 0.05; ^*a*^ vs. the LPS group, *P* < 0.05; ^*b*^ vs. the LPS + sham group, *P* < 0.05; ^*&*^ vs. the ICH group, *P* < 0.05; ns: no significance

Endogenous expression of CCR5 in the brain on the side of the hematoma was evaluated via Western blotting at 1, 3, and 7 d after ICH and LPS administration. The expression of CCR5 exhibited an upward trend and peaked at 3 days post-ICH (Fig. 6B, C). The levels of CCL5 in the brain and periphery were assessed via ELISA, and alterations similar to those in CCR5 expression were observed in the ipsilateral hemisphere (Fig. 6D, E). Therefore, we further investigated the levels of CCL5 in the brain and periphery in the sham, LPS, LPS + sham, ICH, and ICH + LPS groups at 3 days after ICH. The level of endogenous CCL5 was significantly increased in the ipsilateral brain and the peripheral blood in the ICH + LPS group compared with the ICH group (*P* < 0.05; Fig. 6F, G). Double immunostaining showed that CCR5 was mainly colocalized with GFAP (an astrocyte marker), MPO (a monocyte marker), and IBA-1 (a microglial marker) (Fig. 6A). The relative increase in the number of MPO^+^ cells suggested that central inflammation around the lesion area was exacerbated in the ICH + LPS group (Fig. 6A).

### Severe peripheral inflammation promoted the CCR5/JAK2/STAT3/MMP9 signaling pathway 3 days after ICH

**Fig. 7:**
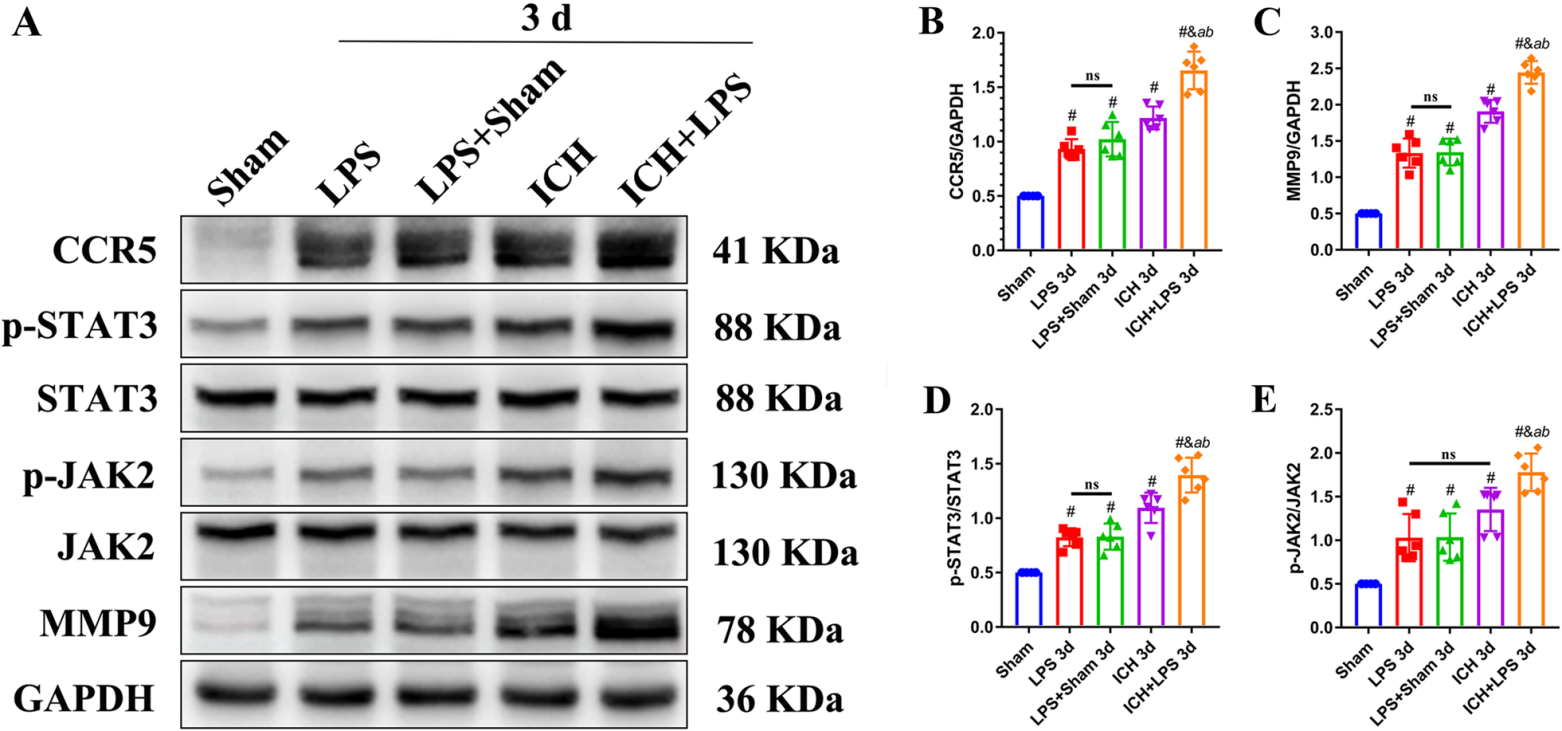
Severe peripheral inflammation promoted the CCR5/JAK2/STAT3/MMP9 signaling pathway 3 days after ICH. **A**–**E** Western blot images and quantitative analysis of CCR5, p-JAK2, p-STAT3, and MMP9 expression in the ipsilateral hemisphere in the sham, LPS, LPS + sham, ICH, and ICH + LPS groups 3 days after ICH. The data are expressed as the mean ± SD. ^#^ vs. the sham group, *P* < 0.05; ^*a*^ vs. the LPS group, *P* < 0.05; ^*b*^ vs. the LPS + sham group, *P* < 0.05; ^*&*^ vs. the ICH group, *P* < 0.05; ns: no significance

Western blotting demonstrated that the expression of CCR5 and MMP9 on the affected brain was significantly upregulated in the ICH + LPS group compared to the ICH group 3 days after ICH (*P* < 0.05; Fig. 7A–C). In addition, the phosphorylation of JAK2 and STAT3 in the brain was markedly elevated in the ICH + LPS group compared with the ICH group 3 days after ICH (*P* < 0.05; Fig. 7D, E).

### MVC- and rCCL5-mediated regulation of CCR5 expression affected BBB integrity and neurobehavioral function via the CCR5/JAK2/STAT3/MMP9 signaling pathway 3 days after ICH and LPS-induced potentiation of peripheral inflammation

**Fig. 8:**
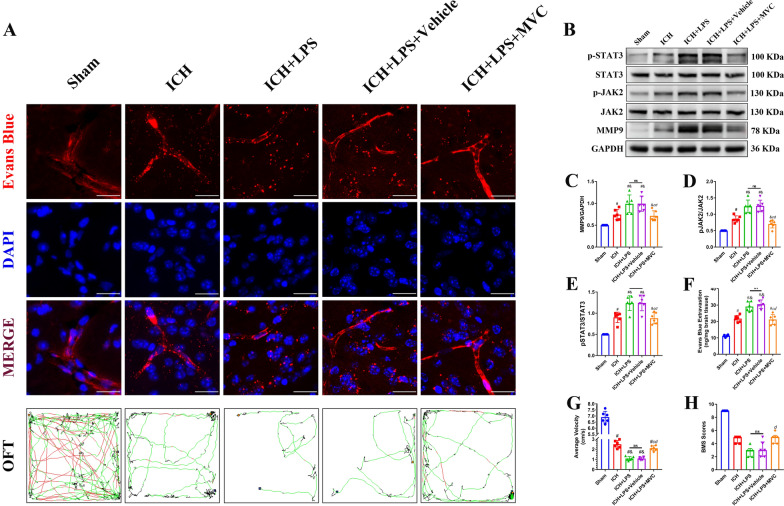
Administration of the specific CCR5 inhibitor MVC alleviated cerebral damage via the JAK2/STAT3/MMP9 signaling pathway 3 days after ICH and potentiation of peripheral inflammation. **A** EB fluorescence images (scale bar = 50 μm) and trajectories in the OFT. According to preset parameters, the mouse trajectories are presented in three colors: black (low speed), green (middle speed), and red (high speed). **B**–**E** Western blot images and quantitative analysis of p-JAK2, p-STAT3, and MMP9 levels in the ipsilateral hemisphere in the sham, ICH, ICH + LPS, ICH + LPS + vehicle, and ICH + LPS + MVC groups 3 days after ICH. (G, H) Statistical analysis of average velocity and BMS scores. The data are expressed as the mean ± SD. ^#^ vs. the sham group, *P* < 0.05; ^*c*^ vs. the ICH + LPS group, *P* < 0.05; ^*d*^ vs. the ICH + LPS + vehicle group, *P* < 0.05; ^*&*^ vs. the ICH group, *P* < 0.05; ns: no significance

**Fig. 9:**
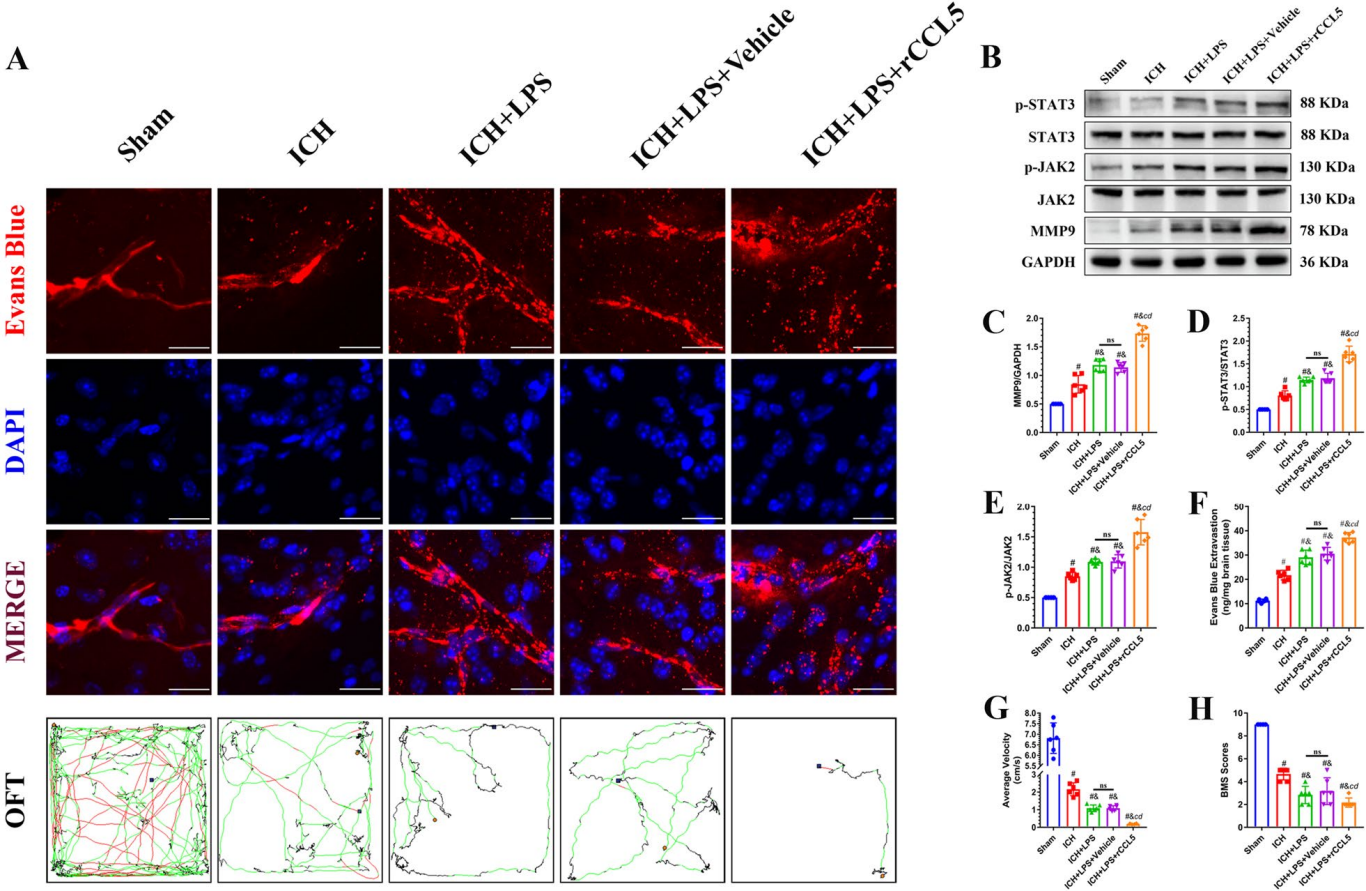
Administration of the specific CCR5 agonist rCCL5 exacerbated cerebral damage via the JAK2/STAT3/MMP9 signaling pathway 3 days after ICH and induction of severe peripheral inflammation. **A** EB fluorescence images (scale bar = 50 μm) and trajectories in the OFT. According to the preset parameters, the mouse trajectories are presented in three colors: black (low speed), green (middle speed), and red (high speed). **B**–**E** Western blot images and quantitative analysis of p-JAK2, p-STAT3, and MMP9 levels in the ipsilateral hemisphere in the sham, ICH, ICH + LPS, ICH + LPS + vehicle, and ICH + LPS + rCCL5 groups 3 days after ICH. **G**, **H** Statistical analysis of average velocity and BMS scores. The data are expressed as the mean ± SD. ^#^ vs. the sham group, *P* < 0.05; ^*c*^ vs. the ICH + LPS group, *P* < 0.05; ^*d*^ vs. the ICH + LPS + vehicle group, *P* < 0.05; ^*&*^ vs. the ICH group, *P* < 0.05; ns: no significance

BBB integrity and neurobehavioral function were evaluated via the EB extravasation assay, BMS scale, and OFT. EB extravasation in the ipsilateral hemisphere was significantly increased and BMS scores and the average velocity were markedly reduced in the ICH + LPS + Vehicle group compared with the sham and ICH groups (*P* < 0.05; Fig. 8A, F–H). However, the degree of EB extravasation on the affected side, the average velocity and BMS scores were decreased in the ICH + LPS + MVC group compared with the ICH + LPS + Vehicle group (*P* < 0.05; Fig. 8A, F–H). Conversely, rCCL5 administration significantly increased the leakage of EB in the ipsilateral hemisphere and aggravated neurobehavioral dysfunction after ICH induction and LPS injection (*P* < 0.05; Fig. 9A, F–H).

Moreover, the expression levels of factors downstream of CCR5, including p-JAK2, p-STAT3, and MMP9, were assessed via western blotting. The results suggested that the expression levels of p-JAK2, p-STAT3, and MMP9 were significantly reduced in the ICH + LPS + MVC group compared with the ICH + LPS + vehicle group (*P* < 0.05; Fig. 8B–E). The mice in the ICH + LPS + rCCL5 group showed upregulated expression of p-JAK2, p-STAT3, and MMP9 (*P* < 0.05; Fig. 9B–E). This further proved, to some degree, that the CCR5/JAK2/STAT3/MMP9 signaling pathway participates in CCR5-mediated BBB disruption and locomotor dysfunction.

### CRISPR-mediated JAK2 knockdown partly reversed rCCL5-induced BBB disruption and neurobehavioral dysfunction by inhibiting the JAK2/STAT3/MMP9 pathway 3 days after ICH and LPS-induced potentiation of peripheral inflammation

**Fig. 10:**
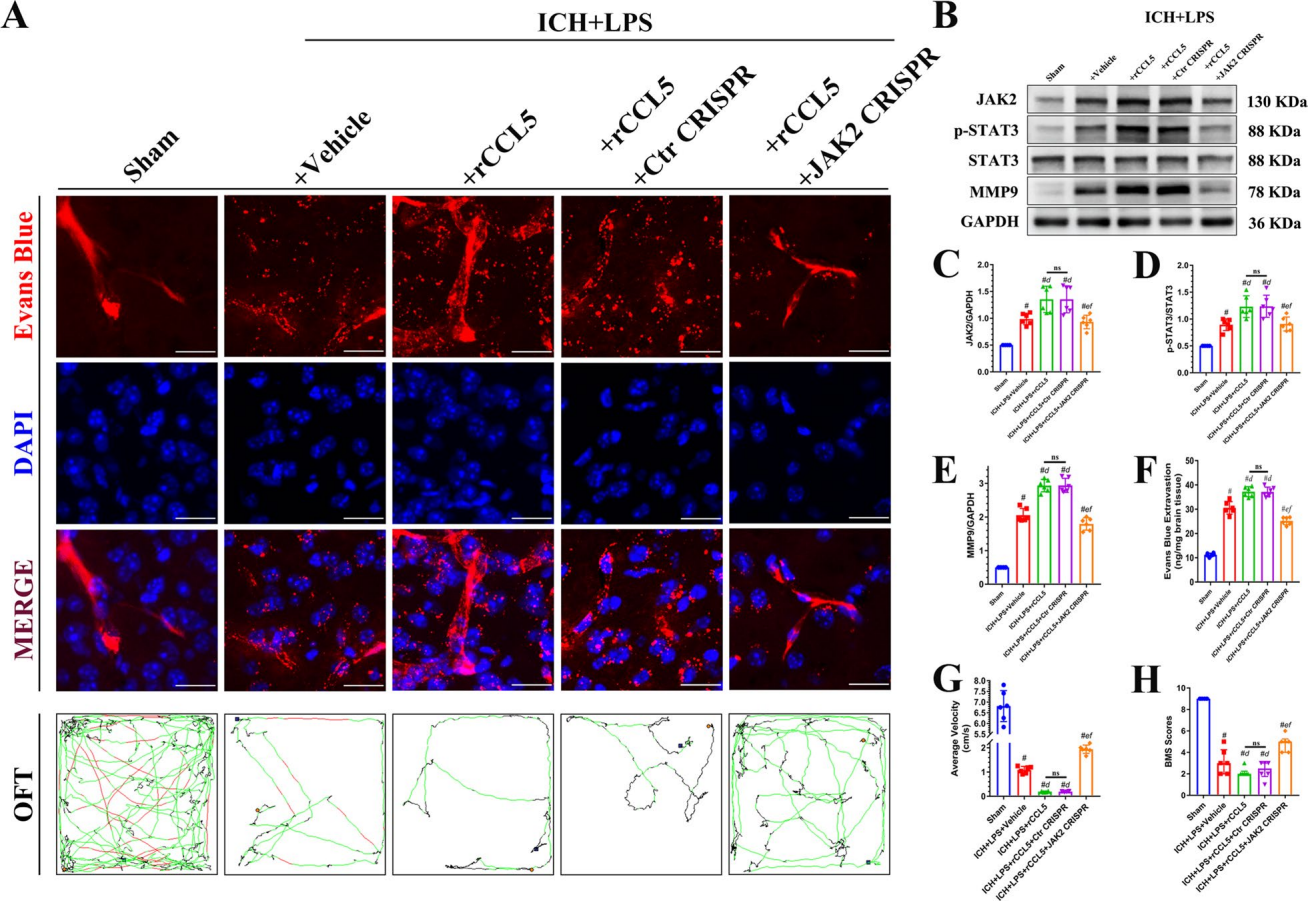
CRISPR-mediated JAK2 knockdown reversed cerebral damage induced by severe peripheral inflammation 3 days after ICH via inhibition of the JAK2/STAT3/MMP9 signaling pathway. **A** EB fluorescence images (scale bar = 50 μm) and trajectories in the OFT. According to preset parameters, the mouse trajectories are presented in three colors: black (low speed), green (middlzsxxdsse speed), and red (high speed). **B**–**E** Western blot images and quantitative analysis of JAK2, p-STAT3, and MMP9 levels in the ipsilateral hemisphere in the sham, ICH + LPS + vehicle, ICH + LPS + rCCL5, ICH + LPS + rCCL5 + Ctr CRISPR, and ICH + LPS + rCCL5 + JAK2 CRISPR groups 3 days after ICH. **G**, **H**) Statistical analysis of average velocity and BMS scores. The data are expressed as the mean ± SD. ^#^ vs. the sham group, *P* < 0.05; ^*d*^ vs. the ICH + LPS + vehicle group, *P* < 0.05; ^*e*^ vs. the ICH + LPS + rCCL5 group, *P* < 0.05; ^*f*^ vs. the ICH + LPS + rCCL5 + Ctr CRISPR group, *P* < 0.05; ns: no significance. Ctr: control

Before intervention, we evaluated the JAK2 knockdown efficiency of CRISPR plasmid with western blot (Additional file 1: Fig. S5). Our results showed that the expression of JAK2 in the sham + JAK2 CRISPR group was significantly inhibited and lower than that of the mice in the sham and sham + Ctr CRISPR group (Additional file 1: Fig. S5A). Mice in the ICH + LPS + rCCL5 + JAK2 CRISPR group were pretreated with a JAK2 CRISPR plasmid, and JAK2 downregulation was confirmed by Western blot analysis (Fig. 10B, C). The phosphorylation of STAT3 and the expression of MMP9 in the ICH + LPS + rCCL5 + JAK2 CRISPR group were significantly lower than those in the ICH + LPS + rCCL5 + Ctr CRISPR group (*P* < 0.05; Fig. 10B, D, E). rCCL5-induced loss of BBB integrity and impairment of neurobehavioral function were partly reversed by CRISPR-mediated JAK2 knockdown, as indicated by the comparison of EB extravasation, BMS scores, and OFT performance between the ICH + LPS + rCCL5 + JAK2 CRISPR and ICH + LPS + rCCL5 + Ctr CRISPR groups. The degree of EB extravasation was significantly reduced in the ICH + LPS + rCCL5 + JAK2 CRISPR group compared to the ICH + LPS + rCCL5 + Ctr CRISPR group (*P* < 0.05; Fig. 10A, F). In addition, BMS scores and the average velocity were significantly increased in the ICH + LPS + rCCL5 + JAK2 CRISPR group compared with the ICH + LPS + rCCL5 + Ctr CRISPR group 3 days after ICH and potentiation of peripheral inflammation (*P* < 0.05; Fig. 10G, H). The above results further confirmed that JAK2 is downstream of CCR5 and partly contributes to CCR5-mediated BBB disruption and locomotor dysfunction.

## Discussion

Secondary inflammatory damage after ICH is an intractable problem, especially in patients who develop infection, for which is still lack of effective intervention measures. The most characteristic outcomes are inflammation and prolonged cerebral edema due to disturbance of environmental homeostasis in the brain resulting from BBB disruption. Studies on environmental homeostasis disruption in the brain have mainly focused on changes in the brain itself; however, in recent years, crosstalk between the periphery and brain has attracted increasing attention from researchers.

In this study, we explored the effects of severe peripheral inflammation on BBB disruption and neurobehavioral impairment after ICH through activation of the CCR5/JAK2/STAT3/MMP9 signaling pathway in mice. The novel findings are as follows: [1] when peripheral inflammation was potentiated after ICH, the expression of CCR5 and its ligand CCL5 was upregulated and peaked at 3 days post-ICH. CCR5 was localized in microglia, astrocytes, and monocytes. (2) The expression of downstream effectors of CCR5, including p-JAK2, p-STAT3 and MMP9, was increased, and thus the expression of tight junction proteins (e.g., ZO-1 and CLDN5) was decreased at 3 days after ICH and LPS administration. (3) Specific inhibition of CCR5 by MVC obviously alleviated BBB disruption and dyskinesia by abating the expression of CCR5/JAK2/STAT3/MMP9 signaling pathway-related proteins in LPS-treated ICH model mice. (4) rCCL5, as a specific CCR5 activator, had the opposite effects as MVC, and these effects were partly reversed by CRISPR-mediated JAK2 knockdown. In addition to these findings, we found that the level of CCL5 in both the ipsilateral hemisphere and peripheral blood was closely associated with the degree of cerebral edema around the hematoma, which is consistent with a previous report [24].

CCR5 is a chemokine receptor that plays a vital role in recruiting and activating inflammatory cells and is widely expressed on multiple cell types, including microglia, astrocytes, and monocytes, in most brain regions and even the blood [12, 13, 32]; this is consistent with our double immunofluorescence results. In addition, it has been reported that CCR5 mRNA and protein levels are increased in the brain in a middle cerebral artery occlusion model [33] and ICH model [12]. Similarly, our findings showed that CCR5 expression was increased, especially at 1 and 3 days after ICH. Moreover, CCR5 expression was further elevated by severe peripheral inflammation induced by LPS i.p. injection. The trend in the expression of the CCR5 ligand CCL5 in the brain and blood exhibited a similar trend as the expression of CCR5 in the brain in the ICH + LPS model and was accompanied by upregulation of p-JAK2, p-STAT3, and MMP9. Previous studies have proven that high CCL5 levels are observed in various models of inflammatory injury and that the increase in CCL5 levels is mainly caused by facilitation of the secretion of several chemokines, including interferon, tumor necrosis factor-α, and IL-6 [34]. These secreted cytokines can be detected in the peripheral nervous system and affect innate immune cells in the brain by providing feedback via neuroimmune circuits [35]. In parallel, CCL5/CCR5 axis potentiation can lead to superfluous infiltration of inflammatory cells into lesions, which promotes widespread molecular crosstalk between central and peripheral immune cells following BBB disruption [36]. Double immunofluorescence also revealed that the number of infiltrated monocytes around the hematoma was increased, although quantitative data is lacking. This means that the increase in CCL5 levels resulting from LPS administration may have been the primary initiator of inflammatory attacks and that p-JAK2 and p-STAT3 subsequently activated by CCR5 signaling and then aggravate MMP9-mediated BBB disruption after ICH.

MVC, as an FDA-approved selective antagonist of CCR5, exerts good anti-inflammatory effects in animal models of autoimmune encephalomyelitis [37] and ICH [12]. It was initially used to impede HIV infection and has been found to improve the outcomes of HIV patients to some degree [14]. However, its effects on ICH combined with infection have not been elucidated. In this study, we deliberately modeled peripheral infection in ICH model mice via i.p. LPS injection and found that the prognoses of these mice were worse than that of untreated ICH model mice. Additionally, we administered MVC by i.p. injection 1 day before, 1 h before, and 1 day after ICH in this study. The results indicated that MVC-mediated inhibition of CCR5 significantly ameliorated BBB disruption and neurological deficits and decreased the expression of downstream proteins, including p-JAK2, p-STAT3, and MMP9. After the onset of ICH in the basal ganglia, perihematomal brain tissues, especially in the striatal region, exhibit severe hematoma and edema. The striatum, a part of the extrapyramidal system, receives inputs from the cerebral cortex and thalamus to regulate muscle tone and coordinate various delicate and complex movements [38]. In this study, behavioral functions were mainly assessed by the OFT and BMS and revealed that ICH mice exhibited neurobehavioral deficits at 1 day, 3 days, and 7 days post-ICH and that these deficits were exacerbates by peripheral infection due to increased neuronal loss in the striatal region [12]. Thus, early inhibition of CCR5 provided constant protection of the BBB and limited the development and progression of cerebral edema, in turn ameliorating sensorimotor dysfunction of ICH + LPS group mice.

**Fig. 11:**
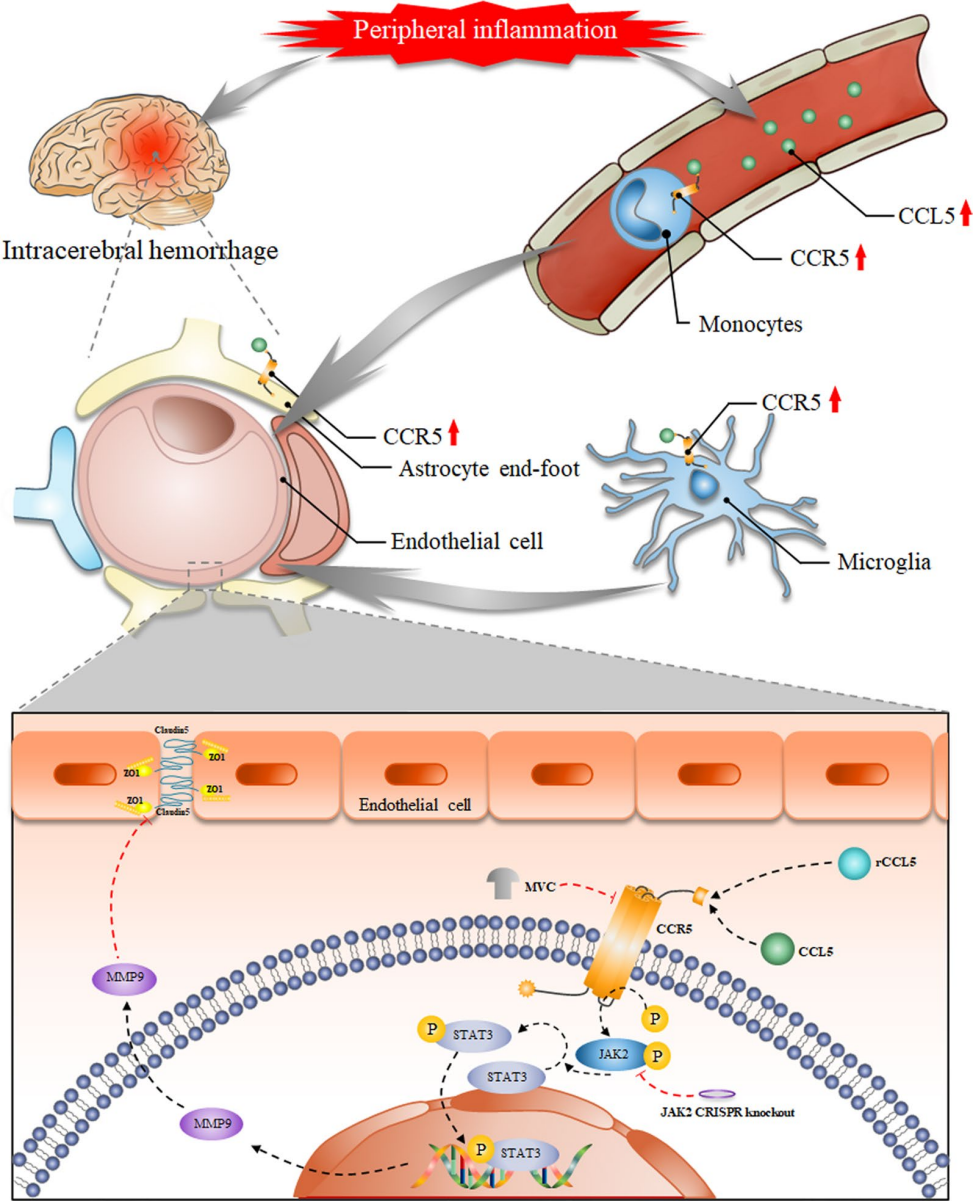
Diagram showing that severe peripheral inflammation increases CCR5 expression in inflammatory cells, including astrocytes, microglia, and monocytes, by elevating the level of CCL5 in the brain and circulating blood. Overexpression of CCR5 accelerates JAK2 phosphorylation and subsequently promotes pSTAT3 production and nuclear transport to facilitate MMP9 secretion. The increase in MMP9 protein expression decreases the expression of tight junction proteins such as ZO-1 and CLDN5, causing loss of BBB integrity. MVC can partly reverse these impairments by specifically inhibiting CCR5 expression, and CRISPR-mediated JAK2 knockdown exerts identical effects by impeding the phosphorylation of downstream proteins. Conversely, rCCL5 disrupts BBB integrity via the specific activation of CCR5

We further induced CCR5 expression in ICH + LPS group mice by administering the specific activator rCCL5 through i.p. injection. The increase in the levels of CCR5 effectors in the circulating blood in ICH + LPS + rCCL5 group mice than ICH + LPS + vehicle group mice promoted the systemic inflammatory cytokine cascade and worsened prognosis. In this study, a large degree of EB extravasation and neurobehavioral deficits accompanied by an increase in the levels of downstream proteins, including p-JAK2, p-STAT3, and MMP9, were observed after rCCL5 administration. Importantly, CRISPR-mediated JAK2 knockdown reversed the detrimental effects of rCCL5 and inhibited the expression of p-STAT3 and MMP9. Collectively, our results support the hypothesis that severe peripheral inflammation further activate the CCL5/CCR5 axis in multiple inflammatory cell types, including microglia, astrocytes, and monocytes, and aggravates BBB disruption and neurobehavioral dysfunction after ICH, possibly in part through the CCR5/JAK2/STAT3 signaling pathway (Fig. 11).

However, there are several limitations to this study. First, CCR5 is widely expressed on multiple cells, and similarly, JAK2 and STAT3 serve as classic inflammatory signal transducers in various kinds of cells [14–16]. Thus, in this study, it was difficult to confirm the cell type that plays a dominant role in the effects of CCR5. Second, this study focused on the effects of CCR5 on BBB integrity after ICH and induction of severe peripheral inflammation. A previous study confirmed that other downstream proteins, such as NF-κB, mediate BBB disruption after CCR5 activation during systemic inflammation [39, 40], and we could not exclude their effects. Third, gene knockout, a robust technique for verifying findings in animals, could not be performed in this study due to certain limitation. Finally, we only used male mice in this study and did not explore sex differences in neuroinflammation and the mechanisms of ICH [41].

## Conclusions

Our study revealed that severe peripheral inflammation further activates the CCL5/CCR5 axis in multiple inflammatory cell types, including microglia, astrocytes, and monocytes, and aggravates BBB disruption and neurobehavioral dysfunction after ICH, possibly in part through the JAK2/STAT3 signaling pathway. MVC partly ameliorates these impairments by specifically decreasing CCR5 expression, while rCCL5 exacerbates these impairments by upregulating CCR5 expression. However, CRISPR-mediated JAK2 knockdown partly reverses rCCL5-induced damage. Thus, CCR5 might be a potential target for the clinical treatment of BBB disruption exacerbation due to infection following ICH.

## Supplementary Information

**Additional file 1: Table S1.** Summary of the experimental groups and mortality rate in the study. **Fig. S1.** Serum levels of IL-6 and TNF-α after i.p. injection of LPS. **Fig. S2.** All open field test route record images in this study. **Fig. S3.** Full layers of magnetic resonance imaging in Sham, ICH, and ICH + LPS groups on 3 days post-ICH. **Fig. S4.** Full western blot bands. **Fig. S5.** JAK2 protein expression after JAK2 CRISPR Knockdown of sham group. Data Sets of this study

## Data Availability

All data generated or analysed during this study are included in this published article and its additional information files.

## Abbreviations

ICH: Intracerebral hemorrhage
BBB: Blood‒brain barrier
CCR5: C–C chemokine receptor 5
CCL5: C–C chemokine ligand 5
JAK2: Janus kinase 2
STAT3: Signal transducer and activator of transcription 3
MMP9: Matrix metalloproteinase-9
ZO-1: Zonula occludens-1
CLDN5: Claudin-5
ARRIVE 2.0: Animal Research: Reporting of In Vivo Experiments, Version 2.0
MVC: Maraviroc
LPS: Lipopolysaccharide
OFT: Open field test
BMS: Basso mouse scale
MRI: Magnetic resonance imaging
EB: Evans blue
