# Supplementary material for: CCL5/CCR5-mediated peripheral inflammation exacerbates blood‒brain barrier disruption after intracerebral hemorrhage in mice
Source: J Transl Med. 2023 Mar 14;21:196. doi: 10.1186/s12967-023-04044-3 (PMC10015963; doi:10.1186/s12967-023-04044-3)
Supplement: Supplementary file 1 — Additional file 1: Table S1. Summary of the experimental groups and mortality rate in the study. Fig. S1. Serum levels of IL-6 and TNF-α after i.p. injection of LPS. Fig. S2. All open field test route record images in this study. Fig. S3. Full layers of magnetic resonance imaging in Sham, ICH, and ICH + LPS groups on 3 days post-ICH. Fig. S4. Full western blot bands. Fig. S5. JAK2 protein expression after JAK2 CRISPR Knockdown of sham group. Data Sets of this study [file 12967_2023_4044_MOESM1_ESM.docx]

**Table S1.** Summary of experimental groups and mortality rate in the study.

| **Experimental Groups** | **Behavioral Tests (n=6)** | | | | |  | | **Mortality**  **(%)** | **Subtotal** |
| --- | --- | --- | --- | --- | --- | --- | --- | --- | --- |
|  | **Brain water content**  **/MRI** | **WB**  **/ELISA** | **IF** | **Evans Blue Etravasation** | **Evans Blue Fluorescence** | | **Exclusion** |  |  |
| **Experiment 1** |  |  |  |  |  | |  |  |  |
| Sham | 6 | - | - | 6 | 2 | | 0 | 0 | 14 |
| LPS (day 1, day 3, day 7) | 18 | - | - | 18 | 6 | | 0 | 0 | 42 |
| LPS+Sham (day 1, day 3, day 7) | 18 | - | - | 18 | 6 | | 0 | 0 | 42 |
| ICH (day 1, day 3, day 7) | 18 | - | - | 18 | 6 | | 0 | 2 (4.55%) | 44 |
| ICH+LPS (day 1, day 3, day 7) | 18 | - | - | 18 | 6 | | 0 | 5 (10.64%) | 47 |
| **Experiment 2** |  |  |  |  |  | |  |  |  |
| Sham |  | 6 | 2 |  |  | | 0 | 0 | 8 |
| LPS (day 3) |  | 6 | 2 |  |  | | 0 | 0 | 8 |
| LPS+Sham (day 3) |  | 6 | 2 |  |  | | 0 | 0 | 8 |
| ICH (day 3) |  | 6 | 2 |  |  | | 0 | 0 | 8 |
| ICH+LPS (day 3) |  | 6 | 2 |  |  | | 0 | 0 | 8 |
| **Experiment 3** |  |  |  |  |  | |  |  |  |
| Sham | - | - |  | - | - | | - | - | - |
| ICH | - | - |  | - | - | | - | - | - |
| ICH+LPS | - | - |  | - | - | | - | - | - |
| ICH+LPS+Vehicle | - | - |  | 6 | 2 | | 0 | 1(11.11%) | 9 |
| ICH+LPS+rCCL5 | - | - |  | 6 | 2 | | 0 | 2(20.00%) | 10 |
| ICH+LPS+MVC | - | - |  | 6 | 2 | | 0 | 0 | 8 |
| **Experiment 4** |  |  |  |  |  | |  |  |  |
| Sham | - | - |  | - | - | | - | - | - |
| ICH+LPS+Vehicle | - | - |  | - | - | | - | - | - |
| ICH+LPS+rCCL5 | - | - |  | - | - | | - | - | - |
| ICH+LPS+rCCL5+JAK2 CRISPR | - | - |  | 6 | 2 | | 0 | 0 | 8 |
| ICH+LPS+rCCL5+Ctr CRISPR | - | - |  | 6 | 2 | | 0 | 1(11.11%) | 9 |
| **Total** | 78 | 30 | 10 | 108 | 36 | | 0 | 11(4.03%) | 273 |

ICH, intracerebral hemorrhage; LPS, lipopolysaccharide; MRI, magnetic resonance imaging; WB, western blot; IF, immunofluorescence; MVC, Maraviroc; Ctr, control. “-” means the mice/samples required in this group can be shared with other existing groups.


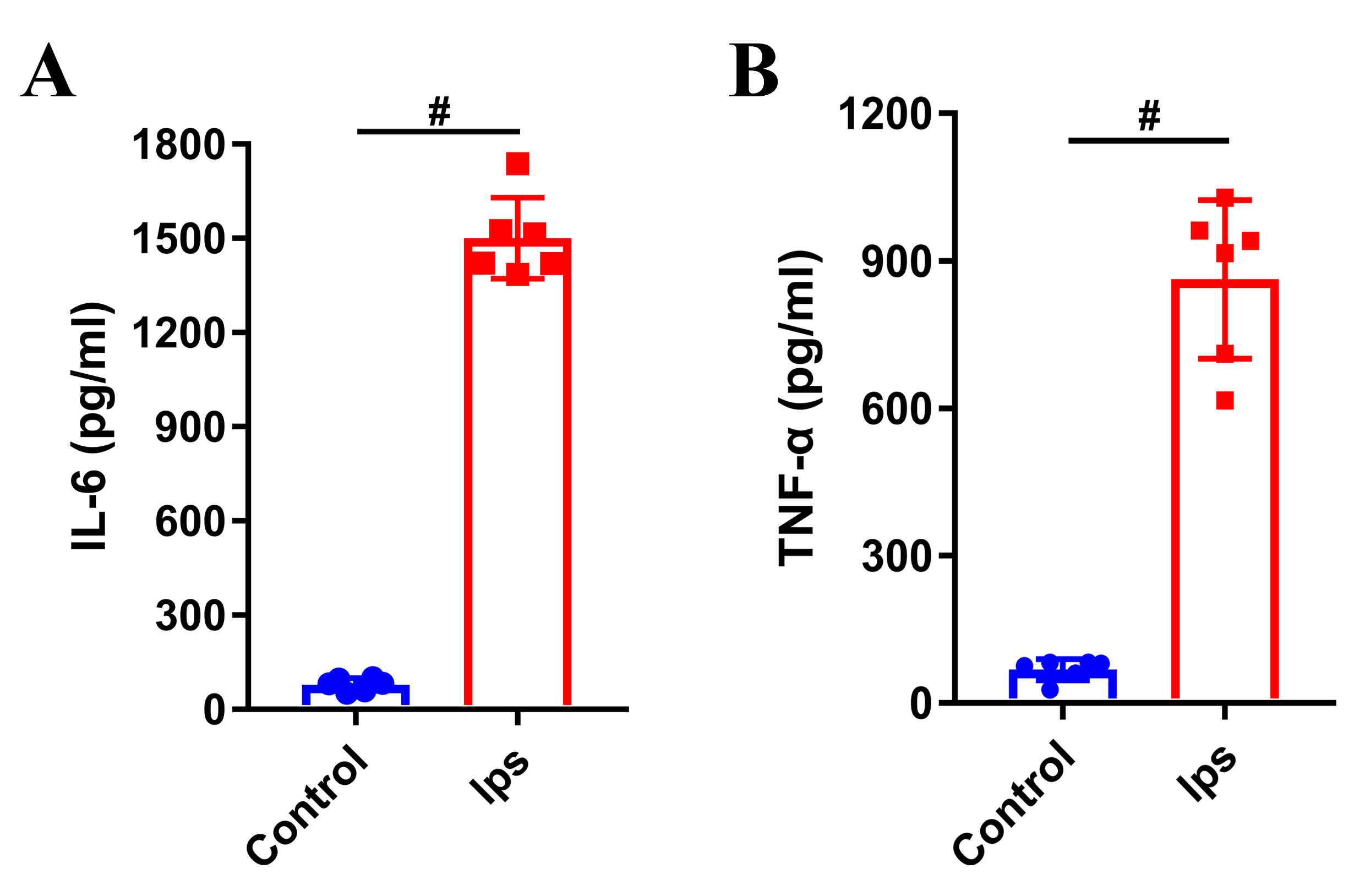


**Fig. S1** The level of interleukin-6 (IL-6) and tumor necrosis factor-α (TNF-α) in serum after intraperitoneal injection of lipopolysaccharide (LPS).


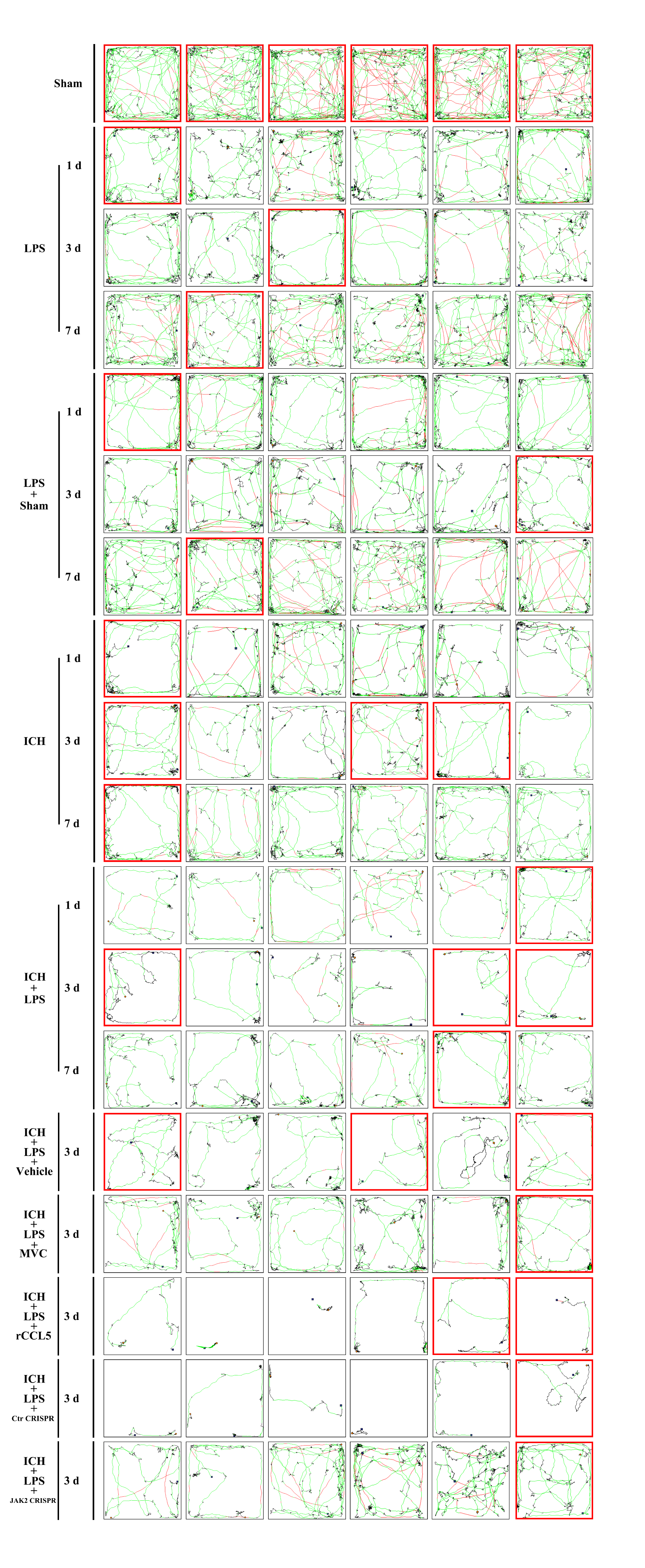


**Fig. S2** All open field test route record images in this study. Red border means the images exhibited in the text. According to the preset parameters, the mouse route track is presented as three colors consisted of black (low speed), green (middle speed), and red (high speed).


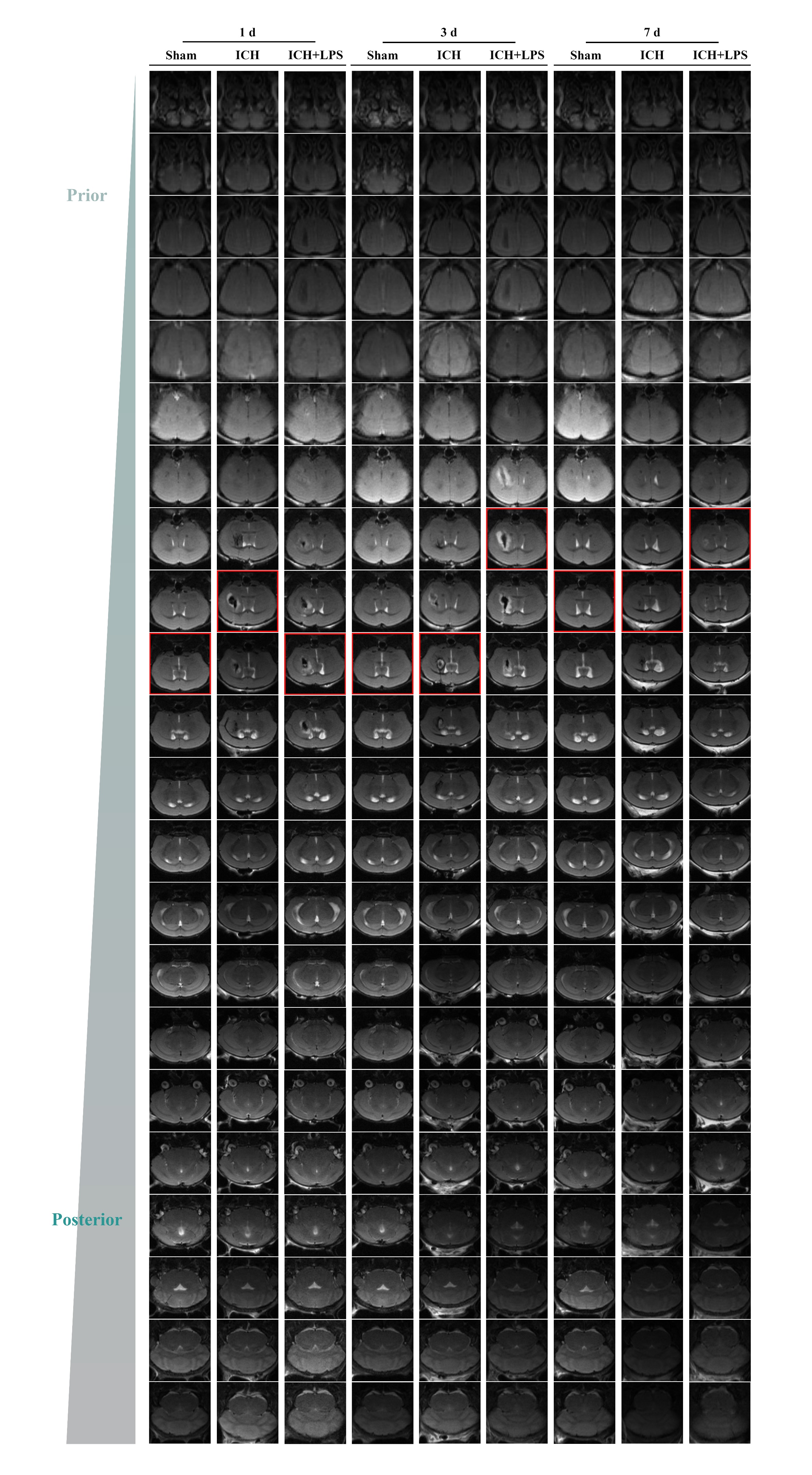


**Fig. S3** Full layers of magnetic resonance imaging in Sham, ICH, and ICH+LPS groups on 3 days post-ICH. Red border means the images exhibited in the text.


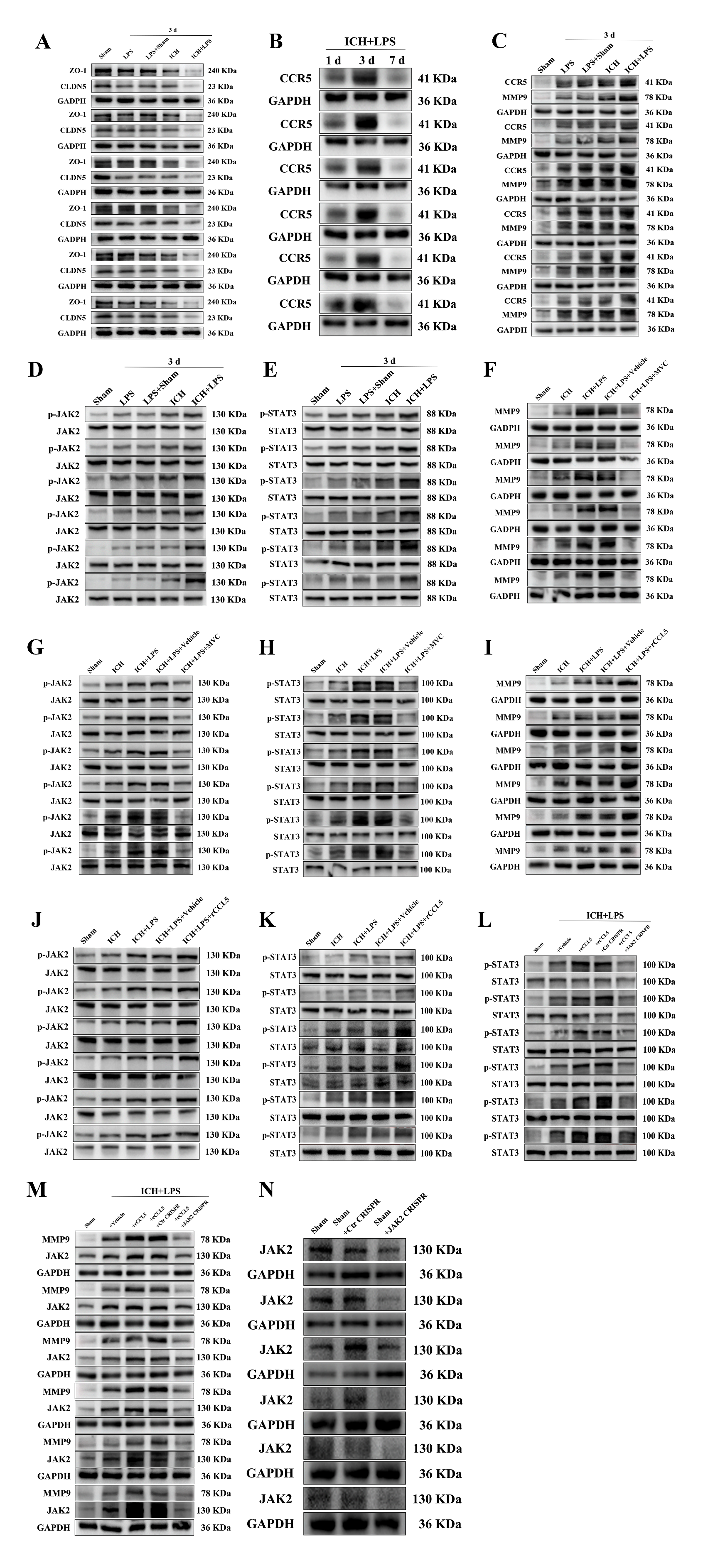


**Fig. S4** Full western blots for (A) ZO-1 and CLDN5 on 3 days, (B) time course of CCR5, (C-E) CCR5, MMP9, p-JAK2, and p-STAT3 levels on 3 days after ICH. (F-K) With the MVC- or rCCL5-mediated regulation, full western blots of MMP9, p-JAK2, and p-STAT3 levels on 3 days after ICH. (L, M) With the JAK2 CRISPR mediated regulation, full western blots of JAK2, MMP9, and p-STAT3 levels on 3 days after ICH. (N) Full western blots for the evaluation of the JAK2 CRISPR knockdown efficiency.


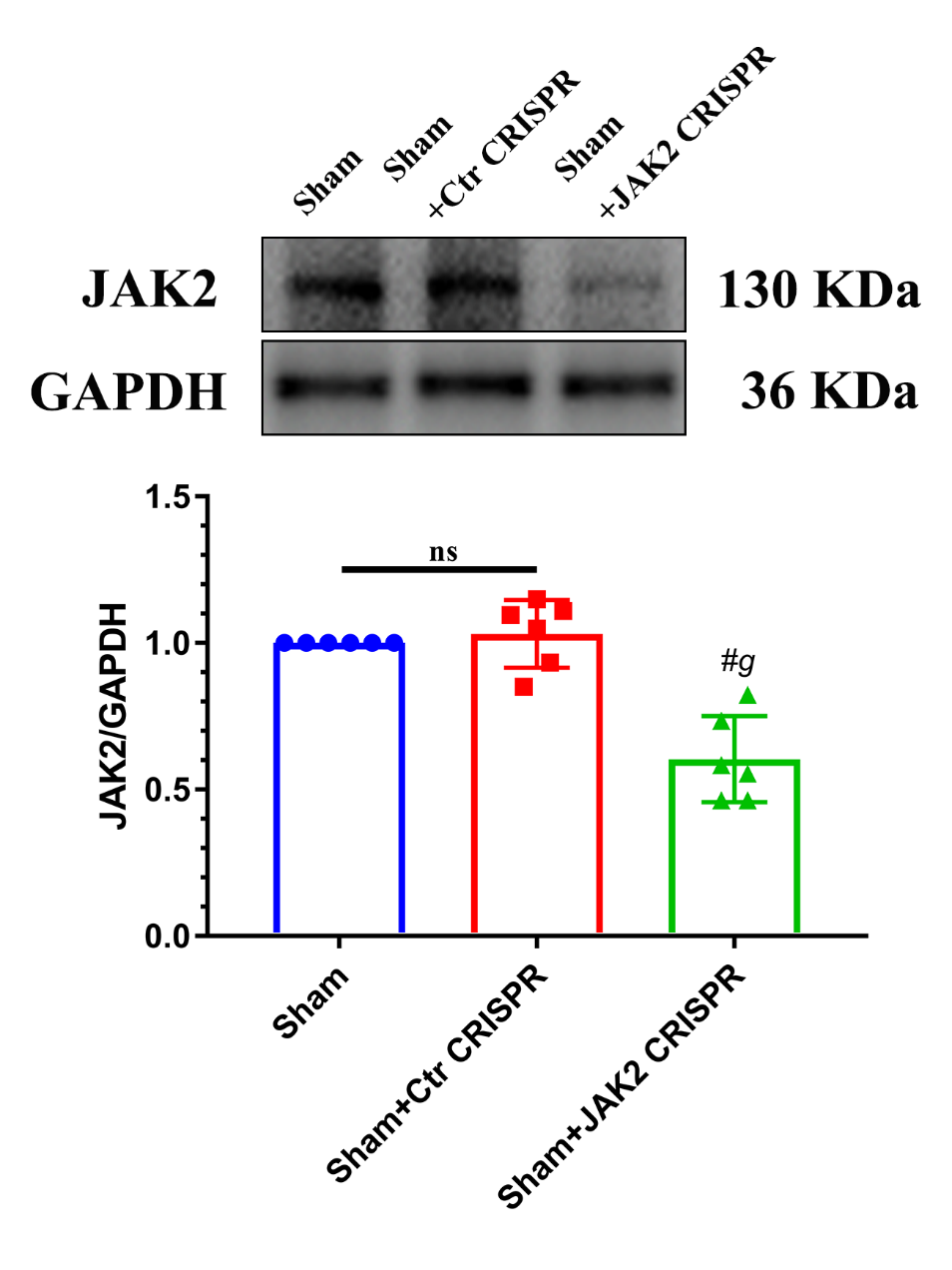


**Fig. S5** JAK2 protein expression after JAK2 CRISPR Knockdown of sham group. ^#^ vs. the sham group, *P*<0.05; ^g^ vs. the sham + Ctr CRISPR group, *P*<0.05; ns: no significance.

**Data Set**

The detailed statistics were applied in the supplement.

**Fig. 2 Intensive peripheral inflammation exacerbated apoplectic dyskinesia on 1, 3, and 7 days after ICH.** (A) Open field test route track and (B, D-F) average velocity statistical analysis in mice. According to the preset parameters, the mouse route track is presented as three colors consisted of black (low speed), green (middle speed), and red (high speed). (C, G-I) Basso mouse scale (BMS) scores statistical analysis in mice. Data were expressed as mean±SD or median with range. ^*^ vs groups on 1 day, *P*<0.05; ^#^ vs sham group, *P*<0.05; *^a^* vs LPS group, *P*<0.05; *^b^* vs LPS+Sham group, *P*<0.05; *^&^* vs ICH group, *P*<0.05; ns: no significance.

**Fig. 2** (B, D-F) Average velocity statistical analysis in mice of Sham, LPS, LPS+Sham, ICH, and ICH+LPS groups on 1 day, 3 days, and 7 days after ICH.

|  | | 1 day | | | | | F(df1, df2)=  value | *P* |
| --- | --- | --- | --- | --- | --- | --- | --- | --- |
|  |  | Sham | LPS | LPS+Sham | ICH | ICH+LPS |  |  |
| Mean (cm/s) | | 6.91 | 5.556 | 5.178 | 4.056 | 2.93 |  |  |
| Std. Deviation | | 0.4748 | 0.6572 | 0.6214 | 0.6496 | 0.4752 |  |  |
| Normality : Shapiro-Wilk normality test | W | 0.9555 | 0.981 | 0.9756 | 0.9113 | 0.8793 |  |  |
|  | *P* | 0.7844 | 0.9563 | 0.9275 | 0.4450 | 0.2657 |  |  |
| Homogeneity of variances test : Levene Statistic | |  |  |  |  |  | F(4, 25)=0.422 | 0.7912 |
|  | | 3 days | | | | | F(df1, df2)=  value | *P* |
|  |  | Sham | LPS | LPS+Sham | ICH | ICH+LPS |  |  |
| Mean (cm/s) | | 6.911 | 4.773 | 4.739 | 2.499 | 1.09 |  |  |
| Std. Deviation | | 0.4745 | 0.367 | 0.3968 | 0.3674 | 0.1875 |  |  |
| Normality : Shapiro-Wilk normality test | W | 0.9551 | 0.9485 | 0.9392 | 0.9636 | 0.9524 |  |  |
|  | *P* | 0.7814 | 0.7281 | 0.6529 | 0.8468 | 0.7600 |  |  |
| Homogeneity of variances test : Levene Statistic | |  |  |  |  |  | F(4, 25)=1.372 | 0.2719 |
|  | | 7 days | | | | | F(df1, df2)=  value | *P* |
|  |  | Sham | LPS | LPS+Sham | ICH | ICH+LPS |  |  |
| Mean (cm/s) | | 6.911 | 6.137 | 6.077 | 5.17 | 3.916 |  |  |
| Std. Deviation | | 0.4745 | 0.5841 | 0.3537 | 0.5748 | 0.5635 |  |  |
| Normality : Shapiro-Wilk normality test | W | 0.9551 | 0.9757 | 0.8729 | 0.8736 | 0.9533 |  |  |
|  | *P* | 0.7814 | 0.9281 | 0.2380 | 0.2411 | 0.7671 |  |  |
| Homogeneity of variances test : Levene Statistic | |  |  |  |  |  | F(4, 25)=0.4443 | 0.7755 |

| 1 day | One way ANOVA | Tukey's multiple comparisons test  *P* value |
| --- | --- | --- |
| Sham vs. LPS 1d |  | 0.0038 |
| Sham vs. LPS+Sham 1d |  | 0.0002 |
| Sham vs. ICH 1d |  | <0.0001 |
| Sham vs. ICH+LPS 1d |  | <0.0001 |
| LPS 1d vs. LPS+Sham 1d |  | 0.7912 |
| LPS 1d vs. ICH 1d |  | 0.0013 |
| LPS 1d vs. ICH+LPS 1d |  | <0.0001 |
| LPS+Sham 1d vs. ICH 1d |  | 0.0202 |
| LPS+Sham 1d vs. ICH+LPS 1d |  | <0.0001 |
| ICH 1d vs. ICH+LPS 1d |  | 0.0195 |
| F | 40.53 |  |
| *P* | <0.0001 |  |
| 3 days | One way ANOVA | Tukey's multiple comparisons test  *P* value |
| Sham vs. LPS 3d |  | <0.0001 |
| Sham vs. LPS+Sham 3d |  | <0.0001 |
| Sham vs. ICH 3d |  | <0.0001 |
| Sham vs. ICH+LPS 3d |  | <0.0001 |
| LPS 3d vs. LPS+Sham 3d |  | 0.9998 |
| LPS 3d vs. ICH 3d |  | <0.0001 |
| LPS 3d vs. ICH+LPS 3d |  | <0.0001 |
| LPS+Sham 3d vs. ICH 3d |  | <0.0001 |
| LPS+Sham 3d vs. ICH+LPS 3d |  | <0.0001 |
| ICH 3d vs. ICH+LPS 3d |  | <0.0001 |
| F | 221.9 |  |
| *P* | <0.0001 |  |
| 7 days | One way ANOVA | Tukey's multiple comparisons test  *P* value |
| Sham vs. LPS 7d |  | 0.1026 |
| Sham vs. LPS+Sham 7d |  | 0.0683 |
| Sham vs. ICH 7d |  | <0.0001 |
| Sham vs. ICH+LPS 7d |  | <0.0001 |
| LPS 7d vs. LPS+Sham 7d |  | 0.9996 |
| LPS 7d vs. ICH 7d |  | 0.0257 |
| LPS 7d vs. ICH+LPS 7d |  | <0.0001 |
| LPS+Sham 7d vs. ICH 7d |  | 0.0402 |
| LPS+Sham 7d vs. ICH+LPS 7d |  | <0.0001 |
| ICH 7d vs. ICH+LPS 7d |  | 0.0025 |
| F | 29.38 |  |
| *P* | <0.0001 |  |
| Total | Two way ANOVA | Dunnett's multiple comparisons test  *P* value |
| Sham |  |  |
| 1 d vs. 3 d |  | >0.9999 |
| 1 d vs. 7 d |  | >0.9999 |
| LPS |  |  |
| 1 d vs. 3 d |  | 0.0051 |
| 1 d vs. 7 d |  | 0.0424 |
| LPS+sham |  |  |
| 1 d vs. 3 d |  | 0.1451 |
| 1 d vs. 7 d |  | 0.0013 |
| ICH |  |  |
| 1 d vs. 3 d |  | <0.0001 |
| 1 d vs. 7 d |  | <0.0001 |
| ICH+LPS |  |  |
| 1 d vs. 3 d |  | <0.0001 |
| 1 d vs. 7 d |  | 0.0004 |
| F | 110.4 |  |
| *P* | <0.0001 |  |

**Fig. 2** (C, G-I) Basso mouse scale (BMS) scores statistical analysis in mice of Sham, LPS, LPS+Sham, ICH, and ICH+LPS groups on 1 day, 3 days, and 7 days after ICH.

|  | 1 day | | | | |
| --- | --- | --- | --- | --- | --- |
|  | Sham | LPS | LPS+Sham | ICH | ICH+LPS |
| Median | 9 | 8 | 8.5 | 7 | 4 |
| 25% Percentile | 9 | 8 | 8 | 6 | 4 |
| 75% Percentile | 9 | 9 | 9 | 7.25 | 5 |
|  | 3 days | | | | |
|  | Sham | LPS | LPS+Sham | ICH | ICH+LPS |
| Median | 9 | 8 | 7.5 | 5 | 3 |
| 25% Percentile | 9 | 7 | 7 | 4 | 2 |
| 75% Percentile | 9 | 8 | 8 | 5 | 3.25 |
|  | 7 days | | | | |
|  | Sham | LPS | LPS+Sham | ICH | ICH+LPS |
| Median | 9 | 8 | 8 | 7 | 6 |
| 25% Percentile | 9 | 7.75 | 7 | 6.75 | 4.75 |
| 75% Percentile | 9 | 9 | 8.25 | 7.25 | 6.25 |

| 1 day | Kruskal-Wallis test | Dunn's multiple comparisons test  *P* value | Mann Whitney test  *P* value (U) |
| --- | --- | --- | --- |
| Sham vs. Lps 1d |  | 0.7841 |  |
| Sham vs. Lps+sham 1d |  | >0.9999 |  |
| Sham vs. ICH 1d |  | 0.0093 |  |
| Sham vs. ICH+lps 1d |  | <0.0001 |  |
| F | 24.69 |  |  |
| *P* | <0.0001 |  |  |
| Sham vs. LPS 1d |  | 0.0640 |  |
| Sham vs. LPS+Sham 1d |  | 0.2528 |  |
| LPS 1d vs. LPS+Sham 1d |  | >0.9999 |  |
| K | 5.74 |  |  |
| *P* | 0.0950 |  |  |
| ICH+LPS 1d vs. ICH 1d |  |  | 0.0022 (0) |
| 3 days | Kruskal-Wallis test | Dunn's multiple comparisons test  *P* value | Mann Whitney test  *P* value |
| Sham vs. Lps 3d |  | 0.3587 |  |
| Sham vs. Lps+sham 3d |  | 0.2315 |  |
| Sham vs. ICH 3d |  | 0.0011 |  |
| Sham vs. ICH+lps 3d |  | <0.0001 |  |
| K | 27.15 |  |  |
| *P* | <0.0001 |  |  |
| Sham vs. LPS 3d |  | 0.0101 |  |
| Sham vs. LPS+Sham 3d |  | 0.0032 |  |
| LPS 3d vs. LPS+Sham 3d |  | >0.9999 |  |
| K | 12.96 |  |  |
| *P* | 0.0002 |  |  |
| ICH+LPS 3d vs. ICH 3d |  |  | 0.0065 (1) |
| 7 days | Kruskal-Wallis test | Dunn's multiple comparisons test  *P* value | Mann Whitney test  *P* value (U) |
| Sham vs. LPS 7d |  | 0.7262 |  |
| Sham vs. LPS+Sham 7d |  | 0.2514 |  |
| Sham vs. ICH 7d |  | 0.0079 |  |
| Sham vs. ICH+LPS 7d |  | <0.0001 |  |
| F | 22.32 |  |  |
| *P* | 0.0002 |  |  |
| Sham vs. LPS 7d |  | 0.1239 |  |
| Sham vs. LPS+Sham 7d |  | 0.0179 |  |
| LPS 7d vs. LPS+Sham 7d |  | >0.9999 |  |
| K | 8.154 |  |  |
| *P* | 0.0157 |  |  |
| ICH+LPS 7d vs. ICH 7d |  |  | 0.0455 (4.5) |
|  |  |  |  |
| Total | Two way ANOVA | Dunnett's multiple comparisons test  *P* value |  |
| Sham |  |  |  |
| 1 d vs. 3 d |  | >0.9999 |  |
| 1 d vs. 7 d |  | >0.9999 |  |
| LPS |  |  |  |
| 1 d vs. 3 d |  | 0.1192 |  |
| 1 d vs. 7 d |  | 0.8538 |  |
| LPS+sham |  |  |  |
| 1 d vs. 3 d |  | 0.0134 |  |
| 1 d vs. 7 d |  | 0.1192 |  |
| ICH |  |  |  |
| 1 d vs. 3 d |  | <0.0001 |  |
| 1 d vs. 7 d |  | 0.8538 |  |
| ICH+LPS |  |  |  |
| 1 d vs. 3 d |  | 0.0002 |  |
| 1 d vs. 7 d |  | 0.0009 |  |
| F | 34.15 |  |  |
| *P* | <0.0001 |  |  |

**Fig. 3 Intensive peripheral inflammation aggravated encephaledema on 1, 3, and 7 days after ICH.** (A) Coronal magnetic resonance imaging (MRI) with T2 image scans. (B-D) Brain water content statistical analysis for different regions on different time points. Data were expressed as mean±SD. ^#^ vs sham group, *P*<0.05; *^a^* vs LPS group, *P*<0.05; *^b^* vs LPS+Sham group, *P*<0.05; *^&^* vs ICH group, *P*<0.05; ns: no significance.

**Fig. 3** (B-D) Brain water content statistical analysis for different regions in mice of Sham, LPS, LPS+Sham, ICH, and ICH+LPS groups on 1 day, 3 days, and 7 days after ICH.

| Basal ganglia | | 1 day | | | | | F(df1, df2)=  value | *P* |
| --- | --- | --- | --- | --- | --- | --- | --- | --- |
|  |  | Sham | LPS | LPS+Sham | ICH | ICH+LPS |  |  |
| Mean (%) | | 77.3 | 77.55 | 77.66 | 78.61 | 79.15 |  |  |
| Std. Deviation | | 0.5533 | 0.365 | 0.399 | 0.4388 | 0.4667 |  |  |
| Normality : Shapiro-Wilk normality test | W | 0.8497 | 0.9756 | 0.8046 | 0.9231 | 0.8084 |  |  |
|  | *P* | 0.1566 | 0.9277 | 0.0647 | 0.5280 | 0.0698 |  |  |
| Homogeneity of variances test : Levene Statistic | |  |  |  |  |  | F(4, 25)=0.5046 | 0.7327 |
| Basal ganglia | | 3 days | | | | | F(df1, df2)=  value | *P* |
|  |  | Sham | LPS | LPS+Sham | ICH | ICH+LPS |  |  |
| Mean (%) | | 77.3 | 78.16 | 78.25 | 79.97 | 81.07 |  |  |
| Std. Deviation | | 0.5533 | 0.2761 | 0.3699 | 0.3983 | 0.3478 |  |  |
| Normality : Shapiro-Wilk normality test | W | 0.8497 | 0.9113 | 0.8593 | 0.9466 | 0.8393 |  |  |
|  | *P* | 0.1566 | 0.4447 | 0.1869 | 0.7125 | 0.1285 |  |  |
| Homogeneity of variances test : Levene Statistic | |  |  |  |  |  | F(4, 25)=1.098 | 0.3794 |
| Basal ganglia | | 7 days | | | | | F(df1, df2)=  value | *P* |
|  |  | Sham | LPS | LPS+Sham | ICH | ICH+LPS |  |  |
| Mean (%) | | 77.3 | 77.14 | 77.07 | 78.02 | 78.32 |  |  |
| Std. Deviation | | 0.5533 | 0.1818 | 0.1931 | 0.1555 | 0.2239 |  |  |
| Normality : Shapiro-Wilk normality test | W | 0.8497 | 0.9054 | 0.9175 | 0.965 | 0.8172 |  |  |
|  | *P* | 0.1566 | 0.4069 | 0.4875 | 0.8571 | 0.0835 |  |  |
| Homogeneity of variances test : Levene Statistic | |  |  |  |  |  | F(4, 25)=5.155 | 0.0036 |
| Cortex | | 1 day | | | | | F(df1, df2)=  value | *P* |
|  |  | Sham | LPS | LPS+Sham | ICH | ICH+LPS |  |  |
| Mean (%) | | 78.02 | 78.3 | 78.27 | 78.99 | 79.31 |  |  |
| Std. Deviation | | 0.3357 | 0.4132 | 0.2463 | 0.2285 | 0.4188 |  |  |
| Normality : Shapiro-Wilk normality test | W | 0.9075 | 0.8237 | 0.9964 | 0.9348 | 0.9385 |  |  |
|  | *P* | 0.4201 | 0.0949 | 0.9990 | 0.6179 | 0.6474 |  |  |
| Homogeneity of variances test : Levene Statistic | |  |  |  |  |  | F(4, 25)=0.4444 | 0.7753 |
| Cortex | | 3 days | | | | | F(df1, df2)=  value | *P* |
|  |  | Sham | LPS | LPS+Sham | ICH | ICH+LPS |  |  |
| Mean (%) | | 78.02 | 78.87 | 79.02 | 79.62 | 79.95 |  |  |
| Std. Deviation | | 0.3357 | 0.3508 | 0.1787 | 0.3556 | 0.4089 |  |  |
| Normality : Shapiro-Wilk normality test | W | 0.9075 | 0.9639 | 0.9142 | 0.9283 | 0.922 |  |  |
|  | *P* | 0.4201 | 0.8495 | 0.4644 | 0.5673 | 0.5200 |  |  |
| Homogeneity of variances test : Levene Statistic | |  |  |  |  |  | F(4, 25)=1.256 | 0.3133 |
| Cortex | | 7 days | | | | | F(df1, df2)=  value | *P* |
|  |  | Sham | LPS | LPS+Sham | ICH | ICH+LPS |  |  |
| Mean (%) | | 77.3 | 77.14 | 77.07 | 78.02 | 78.32 |  |  |
| Std. Deviation | | 0.5533 | 0.1818 | 0.1931 | 0.1555 | 0.2239 |  |  |
| Normality : Shapiro-Wilk normality test | W | 0.8497 | 0.9054 | 0.9175 | 0.965 | 0.8172 |  |  |
|  | *P* | 0.1566 | 0.4069 | 0.4875 | 0.8571 | 0.0835 |  |  |
| Homogeneity of variances test : Levene Statistic | |  |  |  |  |  | F(4, 25)=5.155 | 0.0036 |
| Cerebellum | | 1 day | | | | | F(df1, df2)=  value | *P* |
|  |  | Sham | LPS | LPS+Sham | ICH | ICH+LPS |  |  |
| Mean (%) | | 77.08 | 77.16 | 77.18 | 77.38 | 77.76 |  |  |
| Std. Deviation | | 0.1709 | 0.3272 | 0.4104 | 0.5881 | 0.1561 |  |  |
| Normality : Shapiro-Wilk normality test | W | 0.9685 | 0.8853 | 0.8654 | 0.9293 | 0.9192 |  |  |
|  | *P* | 0.8824 | 0.2944 | 0.2085 | 0.5749 | 0.4998 |  |  |
| Homogeneity of variances test : Levene Statistic | |  |  |  |  |  | F(4, 25)=2.167 | 0.1021 |
| Cerebellum | | 3 days | | | | | F(df1, df2)=  value | *P* |
|  |  | Sham | LPS | LPS+Sham | ICH | ICH+LPS |  |  |
| Mean (%) | | 77.08 | 77.14 | 77.18 | 77.15 | 77.58 |  |  |
| Std. Deviation | | 0.1709 | 0.4424 | 0.2749 | 0.4143 | 0.2613 |  |  |
| Normality : Shapiro-Wilk normality test | W | 0.9685 | 0.8991 | 0.8382 | 0.9701 | 0.9888 |  |  |
|  | *P* | 0.8824 | 0.3685 | 0.1259 | 0.8933 | 0.9861 |  |  |
| Homogeneity of variances test : Levene Statistic | |  |  |  |  |  | F(4, 25)=1.603 | 0.2047 |
| Cerebellum | | 7 days | | | | | F(df1, df2)=  value | *P* |
|  |  | Sham | LPS | LPS+Sham | ICH | ICH+LPS |  |  |
| Mean (%) | | 77.08 | 77.07 | 77.03 | 76.94 | 77.12 |  |  |
| Std. Deviation | | 0.1709 | 0.268 | 0.2749 | 0.2086 | 0.2769 |  |  |
| Normality : Shapiro-Wilk normality test | W | 0.9685 | 0.9528 | 0.9651 | 0.9516 | 0.9046 |  |  |
|  | *P* | 0.8824 | 0.7627 | 0.8577 | 0.7535 | 0.4015 |  |  |
| Homogeneity of variances test : Levene Statistic | |  |  |  |  |  | F(4, 25)=0.1962 | 0.9381 |
| Hippocampus | | 1 day | | | | | F(df1, df2)=  value | *P* |
|  |  | Sham | LPS | LPS+Sham | ICH | ICH+LPS |  |  |
| Mean (%) | | 77.25 | 78.03 | 77.79 | 78.14 | 78.45 |  |  |
| Std. Deviation | | 0.3071 | 0.2793 | 0.3815 | 0.2989 | 0.3184 |  |  |
| Normality : Shapiro-Wilk normality test | W | 0.846 | 0.9714 | 0.9654 | 0.8819 | 0.9559 |  |  |
|  | *P* | 0.1461 | 0.9017 | 0.8605 | 0.2780 | 0.7877 |  |  |
| Homogeneity of variances test : Levene Statistic | |  |  |  |  |  | F(4, 25)=0.2717 | 0.8934 |
| Hippocampus | | 3 days | | | | | F(df1, df2)=  value | *P* |
|  |  | Sham | LPS | LPS+Sham | ICH | ICH+LPS |  |  |
| Mean (%) | | 77.25 | 78.06 | 77.88 | 78.88 | 79.87 |  |  |
| Std. Deviation | | 0.3071 | 0.2618 | 0.3665 | 0.4271 | 0.4153 |  |  |
| Normality : Shapiro-Wilk normality test | W | 0.846 | 0.7803 | 0.9855 | 0.9742 | 0.975 |  |  |
|  | *P* | 0.1461 | 0.0388 | 0.9753 | 0.9196 | 0.9239 |  |  |
| Homogeneity of variances test : Levene Statistic | |  |  |  |  |  | F(4, 25)=0.407 | 0.8018 |
| Hippocampus | | 7 days | | | | | F(df1, df2)=  value | *P* |
|  |  | Sham | LPS | LPS+Sham | ICH | ICH+LPS |  |  |
| Mean (%) | | 77.25 | 77.46 | 77.44 | 77.59 | 77.67 |  |  |
| Std. Deviation | | 0.3071 | 0.3812 | 0.5424 | 0.5405 | 0.4758 |  |  |
| Normality : Shapiro-Wilk normality test | W | 0.846 | 0.9474 | 0.9381 | 0.8596 | 0.9242 |  |  |
|  | *P* | 0.1461 | 0.7196 | 0.6442 | 0.1879 | 0.5358 |  |  |
| Homogeneity of variances test : Levene Statistic | |  |  |  |  |  | F(4, 25)=0.955 | 0.4492 |
| Brainstem | | 1 day | | | | | F(df1, df2)=  value | *P* |
|  |  | Sham | LPS | LPS+Sham | ICH | ICH+LPS |  |  |
| Mean (%) | | 77.33 | 77.38 | 77.38 | 77.53 | 77.56 |  |  |
| Std. Deviation | | 0.3609 | 0.4187 | 0.4534 | 0.4634 | 0.4686 |  |  |
| Normality : Shapiro-Wilk normality test | W | 0.9672 | 0.9178 | 0.8527 | 0.9581 | 0.913 |  |  |
|  | *P* | 0.8730 | 0.4896 | 0.1654 | 0.8048 | 0.4562 |  |  |
| Homogeneity of variances test : Levene Statistic | |  |  |  |  |  | F(4, 25)=0.1841 | 0.9445 |
| Brainstem | | 3 days | | | | | F(df1, df2)=  value | *P* |
|  |  | Sham | LPS | LPS+Sham | ICH | ICH+LPS |  |  |
| Mean (%) | | 77.33 | 77.07 | 77.14 | 77.3 | 77.12 |  |  |
| Std. Deviation | | 0.3609 | 0.2299 | 0.3315 | 0.2129 | 0.2231 |  |  |
| Normality : Shapiro-Wilk normality test | W | 0.9672 | 0.9507 | 0.9049 | 0.9665 | 0.9801 |  |  |
|  | *P* | 0.8730 | 0.7461 | 0.4035 | 0.8681 | 0.9522 |  |  |
| Homogeneity of variances test : Levene Statistic | |  |  |  |  |  | F(4, 25)=0.9849 | 0.4337 |
| Brainstem | | 7 days | | | | | F(df1, df2)=  value | *P* |
|  |  | Sham | LPS | LPS+Sham | ICH | ICH+LPS |  |  |
| Mean (%) | | 77.33 | 76.99 | 77.22 | 77.19 | 77.23 |  |  |
| Std. Deviation | | 0.3609 | 0.1475 | 0.2356 | 0.2735 | 0.2317 |  |  |
| Normality : Shapiro-Wilk normality test | W | 0.9672 | 0.9558 | 0.904 | 0.877 | 0.9367 |  |  |
|  | *P* | 0.8730 | 0.7871 | 0.3982 | 0.2556 | 0.6329 |  |  |
| Homogeneity of variances test : Levene Statistic | |  |  |  |  |  | F(4, 25)=0.987 | 0.4327 |

| Basal ganglia 1 day | One way ANOVA | Tukey's multiple comparisons test  *P* value |
| --- | --- | --- |
| Sham vs. LPS 1d |  | 0.8575 |
| Sham vs. LPS+Sham 1d |  | 0.6288 |
| Sham vs. ICH 1d |  | 0.0003 |
| Sham vs. ICH+LPS 1d |  | <0.0001 |
| LPS 1d vs. LPS+Sham 1d |  | 0.9932 |
| LPS 1d vs. ICH 1d |  | 0.0033 |
| LPS 1d vs. ICH+LPS 1d |  | <0.0001 |
| LPS+Sham 1d vs. ICH 1d |  | 0.0093 |
| LPS+Sham 1d vs. ICH+LPS 1d |  | <0.0001 |
| ICH 1d vs. ICH+LPS 1d |  | 0.2698 |
| F | 18.47 |  |
| *P* | <0.0001 |  |
| Basal ganglia 3 days | One way ANOVA | Tukey's multiple comparisons test  *P* value |
| Sham vs. LPS 3d |  | 0.0081 |
| Sham vs. LPS+Sham 3d |  | 0.0031 |
| Sham vs. ICH 3d |  | <0.0001 |
| Sham vs. ICH+LPS 3d |  | <0.0001 |
| LPS 3d vs. LPS+Sham 3d |  | 0.9948 |
| LPS 3d vs. ICH 3d |  | <0.0001 |
| LPS 3d vs. ICH+LPS 3d |  | <0.0001 |
| LPS+Sham 3d vs. ICH 3d |  | <0.0001 |
| LPS+Sham 3d vs. ICH+LPS 3d |  | <0.0001 |
| ICH 3d vs. ICH+LPS 3d |  | 0.0006 |
| F | 87.99 |  |
| *P* | <0.0001 |  |
| Basal ganglia 7 days | One way ANOVA | Tukey's multiple comparisons test  *P* value |
| Sham vs. LPS 7d |  | 0.8964 |
| Sham vs. LPS+Sham 7d |  | 0.6834 |
| Sham vs. ICH 7d |  | 0.0027 |
| Sham vs. ICH+LPS 7d |  | <0.0001 |
| LPS 7d vs. LPS+Sham 7d |  | 0.9929 |
| LPS 7d vs. ICH 7d |  | 0.0003 |
| LPS 7d vs. ICH+LPS 7d |  | <0.0001 |
| LPS+Sham 7d vs. ICH 7d |  | <0.0001 |
| LPS+Sham 7d vs. ICH+LPS 7d |  | <0.0001 |
| ICH 7d vs. ICH+LPS 7d |  | 0.4235 |
| F | 21.29 |  |
| *P* | <0.0001 |  |
| Cortex 1 day | One way ANOVA | Tukey's multiple comparisons test  *P* value |
| Sham vs. LPS 1d |  | 0.6124 |
| Sham vs. LPS+Sham 1d |  | 0.7101 |
| Sham vs. ICH 1d |  | 0.0004 |
| Sham vs. ICH+LPS 1d |  | <0.0001 |
| LPS 1d vs. LPS+Sham 1d |  | 0.9998 |
| LPS 1d vs. ICH 1d |  | 0.0125 |
| LPS 1d vs. ICH+LPS 1d |  | 0.0002 |
| LPS+Sham 1d vs. ICH 1d |  | 0.0085 |
| LPS+Sham 1d vs. ICH+LPS 1d |  | 0.0001 |
| ICH 1d vs. ICH+LPS 1d |  | 0.4979 |
| F | 15.6 |  |
| *P* | <0.0001 |  |
| Cortex 3 days | One way ANOVA | Tukey's multiple comparisons test  *P* value |
| Sham vs. LPS 3d |  | 0.0014 |
| Sham vs. LPS+Sham 3d |  | 0.0002 |
| Sham vs. ICH 3d |  | <0.0001 |
| Sham vs. ICH+LPS 3d |  | <0.0001 |
| LPS 3d vs. LPS+Sham 3d |  | 0.9468 |
| LPS 3d vs. ICH 3d |  | 0.0059 |
| LPS 3d vs. ICH+LPS 3d |  | <0.0001 |
| LPS+Sham 3d vs. ICH 3d |  | 0.0335 |
| LPS+Sham 3d vs. ICH+LPS 3d |  | 0.0005 |
| ICH 3d vs. ICH+LPS 3d |  | 0.4386 |
| F | 29.75 |  |
| *P* | <0.0001 |  |
| Cortex 7 days | One way ANOVA | Tukey's multiple comparisons test  *P* value |
| Sham vs. LPS 7d |  | 0.8964 |
| Sham vs. LPS+Sham 7d |  | 0.6834 |
| Sham vs. ICH 7d |  | 0.0027 |
| Sham vs. ICH+LPS 7d |  | <0.0001 |
| LPS 7d vs. LPS+Sham 7d |  | 0.9929 |
| LPS 7d vs. ICH 7d |  | 0.0003 |
| LPS 7d vs. ICH+LPS 7d |  | <0.0001 |
| LPS+Sham 7d vs. ICH 7d |  | <0.0001 |
| LPS+Sham 7d vs. ICH+LPS 7d |  | <0.0001 |
| ICH 7d vs. ICH+LPS 7d |  | 0.4235 |
| F | 21.29 |  |
| *P* | <0.0001 |  |
| Cerebellum 1 day | One way ANOVA | Tukey's multiple comparisons test  *P* value |
| Sham vs. LPS 1d |  | 0.9946 |
| Sham vs. LPS+Sham 1d |  | 0.9912 |
| Sham vs. ICH 1d |  | 0.6198 |
| Sham vs. ICH+LPS 1d |  | 0.0280 |
| LPS 1d vs. LPS+Sham 1d |  | >0.9999 |
| LPS 1d vs. ICH 1d |  | 0.8395 |
| LPS 1d vs. ICH+LPS 1d |  | 0.0663 |
| LPS+Sham 1d vs. ICH 1d |  | 0.8642 |
| LPS+Sham 1d vs. ICH+LPS 1d |  | 0.0743 |
| ICH 1d vs. ICH+LPS 1d |  | 0.4091 |
| F | 3.32 |  |
| *P* | 0.0260 |  |
| Cerebellum 3 days | One way ANOVA | Tukey's multiple comparisons test  *P* value |
| Sham vs. LPS 3d |  | 0.9974 |
| Sham vs. LPS+Sham 3d |  | 0.9857 |
| Sham vs. ICH 3d |  | 0.9965 |
| Sham vs. ICH+LPS 3d |  | 0.0959 |
| LPS 3d vs. LPS+Sham 3d |  | 0.9997 |
| LPS 3d vs. ICH 3d |  | >0.9999 |
| LPS 3d vs. ICH+LPS 3d |  | 0.1780 |
| LPS+Sham 3d vs. ICH 3d |  | 0.9998 |
| LPS+Sham 3d vs. ICH+LPS 3d |  | 0.2448 |
| ICH 3d vs. ICH+LPS 3d |  | 0.1866 |
| F | 2.238 |  |
| *P* | 0.0936 |  |
| Cerebellum 7 days | One way ANOVA | Tukey's multiple comparisons test  *P* value |
| Sham vs. LPS 7d |  | >0.9999 |
| Sham vs. LPS+Sham 7d |  | 0.9959 |
| Sham vs. ICH 7d |  | 0.8442 |
| Sham vs. ICH+LPS 7d |  | 0.9987 |
| LPS 7d vs. LPS+Sham 7d |  | 0.9979 |
| LPS 7d vs. ICH 7d |  | 0.8703 |
| LPS 7d vs. ICH+LPS 7d |  | 0.9972 |
| LPS+Sham 7d vs. ICH 7d |  | 0.9647 |
| LPS+Sham 7d vs. ICH+LPS 7d |  | 0.9670 |
| ICH 7d vs. ICH+LPS 7d |  | 0.6987 |
| F | 0.4879 |  |
| *P* | 0.7445 |  |
| Hippocampus 1 day | One way ANOVA | Tukey's multiple comparisons test  *P* value |
| Sham vs. LPS 1d |  | 0.0022 |
| Sham vs. LPS+Sham 1d |  | 0.0515 |
| Sham vs. ICH 1d |  | 0.0005 |
| Sham vs. ICH+LPS 1d |  | <0.0001 |
| LPS 1d vs. LPS+Sham 1d |  | 0.6754 |
| LPS 1d vs. ICH 1d |  | 0.9769 |
| LPS 1d vs. ICH+LPS 1d |  | 0.1904 |
| LPS+Sham 1d vs. ICH 1d |  | 0.3383 |
| LPS+Sham 1d vs. ICH+LPS 1d |  | 0.0111 |
| ICH 1d vs. ICH+LPS 1d |  | 0.4615 |
| F | 11.91 |  |
| *P* | <0.0001 |  |
| Hippocampus 3 days | One way ANOVA | Tukey's multiple comparisons test  *P* value |
| Sham vs. LPS 3d |  | 0.0053 |
| Sham vs. LPS+Sham 3d |  | 0.0401 |
| Sham vs. ICH 3d |  | <0.0001 |
| Sham vs. ICH+LPS 3d |  | <0.0001 |
| LPS 3d vs. LPS+Sham 3d |  | 0.9075 |
| LPS 3d vs. ICH 3d |  | 0.0053 |
| LPS 3d vs. ICH+LPS 3d |  | <0.0001 |
| LPS+Sham 3d vs. ICH 3d |  | 0.0006 |
| LPS+Sham 3d vs. ICH+LPS 3d |  | <0.0001 |
| ICH 3d vs. ICH+LPS 3d |  | 0.0006 |
| F | 46.87 |  |
| *P* | <0.0001 |  |
| Hippocampus 7 days | One way ANOVA | Tukey's multiple comparisons test  *P* value |
| Sham vs. LPS 7d |  | 0.9284 |
| Sham vs. LPS+Sham 7d |  | 0.9505 |
| Sham vs. ICH 7d |  | 0.7107 |
| Sham vs. ICH+LPS 7d |  | 0.5236 |
| LPS 7d vs. LPS+Sham 7d |  | >0.9999 |
| LPS 7d vs. ICH 7d |  | 0.9892 |
| LPS 7d vs. ICH+LPS 7d |  | 0.9340 |
| LPS+Sham 7d vs. ICH 7d |  | 0.9805 |
| LPS+Sham 7d vs. ICH+LPS 7d |  | 0.9080 |
| ICH 7d vs. ICH+LPS 7d |  | 0.9979 |
| F | 0.7245 |  |
| *P* | 0.5835 |  |
| Brainstem 1 day | One way ANOVA | Tukey's multiple comparisons test  *P* value |
| Sham vs. LPS 1d |  | 0.9993 |
| Sham vs. LPS+Sham 1d |  | 0.9993 |
| Sham vs. ICH 1d |  | 0.9271 |
| Sham vs. ICH+LPS 1d |  | 0.8801 |
| LPS 1d vs. LPS+Sham 1d |  | >0.9999 |
| LPS 1d vs. ICH 1d |  | 0.9781 |
| LPS 1d vs. ICH+LPS 1d |  | 0.9537 |
| LPS+Sham 1d vs. ICH 1d |  | 0.9781 |
| LPS+Sham 1d vs. ICH+LPS 1d |  | 0.9537 |
| ICH 1d vs. ICH+LPS 1d |  | >0.9999 |
| F | 0.3289 |  |
| *P* | 0.8559 |  |
| Brainstem 3 days | One way ANOVA | Tukey's multiple comparisons test  *P* value |
| Sham vs. LPS 3d |  | 0.5325 |
| Sham vs. LPS+Sham 3d |  | 0.7675 |
| Sham vs. ICH 3d |  | >0.9999 |
| Sham vs. ICH+LPS 3d |  | 0.6904 |
| LPS 3d vs. LPS+Sham 3d |  | 0.9946 |
| LPS 3d vs. ICH 3d |  | 0.6150 |
| LPS 3d vs. ICH+LPS 3d |  | 0.9989 |
| LPS+Sham 3d vs. ICH 3d |  | 0.8363 |
| LPS+Sham 3d vs. ICH+LPS 3d |  | >0.9999 |
| ICH 3d vs. ICH+LPS 3d |  | 0.7675 |
| F | 1.027 |  |
| *P* | 0.4127 |  |
| Brainstem 7 days | One way ANOVA | Tukey's multiple comparisons test  *P* value |
| Sham vs. LPS 7d |  | 0.2066 |
| Sham vs. LPS+Sham 7d |  | 0.9516 |
| Sham vs. ICH 7d |  | 0.8934 |
| Sham vs. ICH+LPS 7d |  | 0.9679 |
| LPS 7d vs. LPS+Sham 7d |  | 0.5699 |
| LPS 7d vs. ICH 7d |  | 0.6853 |
| LPS 7d vs. ICH+LPS 7d |  | 0.5223 |
| LPS+Sham 7d vs. ICH 7d |  | 0.9997 |
| LPS+Sham 7d vs. ICH+LPS 7d |  | >0.9999 |
| ICH 7d vs. ICH+LPS 7d |  | 0.9988 |
| F | 1.324 |  |
| *P* | 0.2886 |  |

**Fig. 4** **Intensive peripheral inflammation exacerbated blood-brain barrier disruption** **on 1, 3, and 7 days after ICH.** (A, D, G) Evans blue fluorescence images; Scale bar = 50 μm. (C, F, I) Zoom magnified images; The arrows in above zoom magnified images point to the Evans blue dye extravasated from vessels. (B, E, H) Evans blue extravasation quantitative analysis. Data were expressed as mean±SD. ^#^ vs sham group, *P*<0.05; *^a^* vs LPS group, *P*<0.05; *^b^* vs LPS+Sham group, *P*<0.05; *^&^* vs ICH group, *P*<0.05; ns: no significance.

**Fig. 4** (B, E, H) Evans blue extravasation quantitative analysis for mice of Sham, LPS, LPS+Sham, ICH, and ICH+LPS groups on 1 day, 3 days, and 7 days after ICH.

|  | | 1 day | | | | | F(df1, df2)=  value | *P* |
| --- | --- | --- | --- | --- | --- | --- | --- | --- |
|  |  | Sham | LPS | LPS+Sham | ICH | ICH+LPS |  |  |
| Mean (ng/mg) | | 11.23 | 15.82 | 15.66 | 18.03 | 25.4 |  |  |
| Std. Deviation | | 0.6497 | 3.155 | 0.8552 | 1.269 | 1.683 |  |  |
| Normality : Shapiro-Wilk normality test | W | 0.9347 | 0.9363 | 0.9013 | 0.8706 | 0.9348 |  |  |
|  | *P* | 0.6167 | 0.6297 | 0.3814 | 0.2286 | 0.6177 |  |  |
| Homogeneity of variances test : Levene Statistic | |  |  |  |  |  | F(4, 25)= 4.605 | 0.0063 |
|  | | 3 days | | | | | F(df1, df2)=  value | *P* |
|  |  | Sham | LPS | LPS+Sham | ICH | ICH+LPS |  |  |
| Mean (ng/mg) | | 11.23 | 17.42 | 17.15 | 21.74 | 29.12 |  |  |
| Std. Deviation | | 0.6497 | 3.477 | 1.803 | 1.983 | 2.893 |  |  |
| Normality : Shapiro-Wilk normality test | W | 0.9347 | 0.8641 | 0.9463 | 0.9709 | 0.8625 |  |  |
|  | *P* | 0.6167 | 0.2038 | 0.7099 | 0.8982 | 0.1979 |  |  |
| Homogeneity of variances test : Levene Statistic | |  |  |  |  |  | F(4, 25)=5.018 | 0.0041 |
|  | | 7 days | | | | | F(df1, df2)=  value | *P* |
|  |  | Sham | LPS | LPS+Sham | ICH | ICH+LPS |  |  |
| Mean (ng/mg) | | 11.23 | 14.24 | 13.48 | 16.42 | 22.77 |  |  |
| Std. Deviation | | 0.6497 | 1.659 | 1.007 | 2.55 | 1.739 |  |  |
| Normality : Shapiro-Wilk normality test | W | 0.9347 | 0.888 | 0.9012 | 0.8536 | 0.8097 |  |  |
|  | *P* | 0.6167 | 0.3077 | 0.3809 | 0.1683 | 0.0718 |  |  |
| Homogeneity of variances test : Levene Statistic | |  |  |  |  |  | F(4, 25)=1.226 | 0.3249 |

| 1 day | One way ANOVA | Tukey's multiple comparisons test  *P* value |
| --- | --- | --- |
| Sham vs. LPS 1d |  | 0.0012 |
| Sham vs. LPS+Sham 1d |  | 0.0017 |
| Sham vs. ICH 1d |  | <0.0001 |
| Sham vs. ICH+LPS 1d |  | <0.0001 |
| LPS 1d vs. LPS+Sham 1d |  | 0.9999 |
| LPS 1d vs. ICH 1d |  | 0.2221 |
| LPS 1d vs. ICH+LPS 1d |  | <0.0001 |
| LPS+Sham 1d vs. ICH 1d |  | 0.1694 |
| LPS+Sham 1d vs. ICH+LPS 1d |  | <0.0001 |
| ICH 1d vs. ICH+LPS 1d |  | <0.0001 |
| F | 52 |  |
| *P* | <0.0001 |  |
| 3 days | One way ANOVA | Tukey's multiple comparisons test  *P* value |
| Sham vs. LPS 3d |  | 0.0011 |
| Sham vs. LPS+Sham 3d |  | 0.0018 |
| Sham vs. ICH 3d |  | <0.0001 |
| Sham vs. ICH+LPS 3d |  | <0.0001 |
| LPS 3d vs. LPS+Sham 3d |  | 0.9997 |
| LPS 3d vs. ICH 3d |  | 0.0302 |
| LPS 3d vs. ICH+LPS 3d |  | <0.0001 |
| LPS+Sham 3d vs. ICH 3d |  | 0.0193 |
| LPS+Sham 3d vs. ICH+LPS 3d |  | <0.0001 |
| ICH 3d vs. ICH+LPS 3d |  | 0.0001 |
| F | 46.96 |  |
| *P* | <0.0001 |  |
| 7 days | One way ANOVA | Tukey's multiple comparisons test  *P* value |
| Sham vs. LPS 7d |  | 0.0314 |
| Sham vs. LPS+Sham 7d |  | 0.1619 |
| Sham vs. ICH 7d |  | 0.0001 |
| Sham vs. ICH+LPS 7d |  | <0.0001 |
| LPS 7d vs. LPS+Sham 7d |  | 0.9297 |
| LPS 7d vs. ICH 7d |  | 0.1853 |
| LPS 7d vs. ICH+LPS 7d |  | <0.0001 |
| LPS+Sham 7d vs. ICH 7d |  | 0.0371 |
| LPS+Sham 7d vs. ICH+LPS 7d |  | <0.0001 |
| ICH 7d vs. ICH+LPS 7d |  | <0.0001 |
| F | 42.42 |  |
| *P* | <0.0001 |  |

**Fig. 5** **Intensive peripheral inflammation down-regulated tight junction proteins expression on 3 days after ICH.** (A-C) Western blot images and quantitative analysis of zonula occludens-1 (ZO-1) and claudin-5 (CLDN5); Data were expressed as mean±SD. ^#^ vs sham group, *P*<0.05; *^a^* vs LPS group, *P*<0.05; *^b^* vs LPS+Sham group, *P*<0.05; *^&^* vs ICH group, *P*<0.05; ns: no significance. (D, E) Double immunofuorescence staining images and representative zoom in pictures for ZO-1 (green) and CLDN5 (green) in endothelial cells (CD31, red). Scale bar = 50 μm. The arrows in above zoom magnified images point to the significant down-regulated segments of tight junction proteins in the vessels.

**Fig. 5** (A-C) Western blot images and quantitative analysis of zonula occludens-1 (ZO-1) and claudin-5 (CLDN5) for mice of Sham, LPS, LPS+Sham, ICH, and ICH+LPS groups on 3 days after ICH.

| ZO-1 | | 3 day | | | | | F(df1, df2)=  value | *P* |
| --- | --- | --- | --- | --- | --- | --- | --- | --- |
|  |  | Sham | LPS | LPS+Sham | ICH | ICH+LPS |  |  |
| Mean | | 1 | 0.8656 | 0.85 | 0.6151 | 0.3563 |  |  |
| Std. Deviation | | 0 | 0.06919 | 0.07605 | 0.1047 | 0.1241 |  |  |
| Normality : Shapiro-Wilk normality test | W |  | 0.8528 | 0.965 | 0.8376 | 0.8674 |  |  |
|  | *P* |  | 0.1659 | 0.8574 | 0.1246 | 0.2159 |  |  |
| Homogeneity of variances test : Levene Statistic | |  |  |  |  |  | F(4, 25)=2.506 | 0.0677 |
| CLDN5 | | 3 days | | | | | F(df1, df2)=  value | *P* |
|  |  | Sham | LPS | LPS+Sham | ICH | ICH+LPS |  |  |
| Mean | | 1 | 0.6681 | 0.6569 | 0.6653 | 0.3675 |  |  |
| Std. Deviation | | 0 | 0.07914 | 0.06697 | 0.1122 | 0.07119 |  |  |
| Normality : Shapiro-Wilk normality test | W |  | 0.9437 | 0.9739 | 0.9382 | 0.9152 |  |  |
|  | *P* |  | 0.6889 | 0.9178 | 0.6447 | 0.4713 |  |  |
| Homogeneity of variances test : Levene Statistic | |  |  |  |  |  | F(4, 25)=1.72 | 0.1771 |

| ZO-1 | One way ANOVA | Tukey's multiple comparisons test  *P* value |
| --- | --- | --- |
| Sham vs. LPS 3d |  | 0.0810 |
| Sham vs. LPS+Sham 3d |  | 0.0414 |
| Sham vs. ICH 3d |  | <0.0001 |
| Sham vs. ICH+LPS 3d |  | <0.0001 |
| LPS 3d vs. LPS+Sham 3d |  | 0.9977 |
| LPS 3d vs. ICH 3d |  | 0.0003 |
| LPS 3d vs. ICH+LPS 3d |  | <0.0001 |
| LPS+Sham 3d vs. ICH 3d |  | 0.0007 |
| LPS+Sham 3d vs. ICH+LPS 3d |  | <0.0001 |
| ICH 3d vs. ICH+LPS 3d |  | 0.0002 |
| F | 52.46 |  |
| *P* | <0.0001 |  |
| CLDN5 | One way ANOVA | Tukey's multiple comparisons test  *P* value |
| Sham vs. LPS 3d |  | <0.0001 |
| Sham vs. LPS+Sham 3d |  | <0.0001 |
| Sham vs. ICH 3d |  | <0.0001 |
| Sham vs. ICH+LPS 3d |  | <0.0001 |
| LPS 3d vs. LPS+Sham 3d |  | 0.9990 |
| LPS 3d vs. ICH 3d |  | >0.9999 |
| LPS 3d vs. ICH+LPS 3d |  | <0.0001 |
| LPS+Sham 3d vs. ICH 3d |  | 0.9997 |
| LPS+Sham 3d vs. ICH+LPS 3d |  | <0.0001 |
| ICH 3d vs. ICH+LPS 3d |  | <0.0001 |
| F | 52.94 |  |
| *P* | <0.0001 |  |

**Fig. 6 Expressions of C-C chemokine receptor type 5 (CCR5) and C-C chemokine ligand type 5 (CCL5/Rantes) after ICH with intensive peripheral inflammation.** (A) Double immunofuorescence staining images and representative zoom in pictures for CCR5 (green) and in astrocytes (GFAP, red, scale bar=50 μm), monocytes (MPO, red, scale bar=50 μm), and microglial cells (IBA-1, red, scale bar=20 μm). (B, C) Western blot images and quantitative analysis of time course of CCR5 in ipsilateral brain of ICH+LPS group. (D, E) Quantitative analysis of time course of CCL5 in ipsilateral brain and serum of ICH+LPS group. (F, G) Quantitative analysis of CCL5 in ipsilateral brain and serum of sham, LPS, LPS+Sham, ICH, and ICH+LPS groups on 3 days after ICH. Data were expressed as mean±SD. ^*^ vs groups on 1 day after ICH, *P*<0.05; ^#^ vs sham group, *P*<0.05; *^a^* vs LPS group, *P*<0.05; *^b^* vs LPS+Sham group, *P*<0.05; *^&^* vs ICH group, *P*<0.05; ns: no significance.

**Fig. 6** (C) Western blot images and quantitative analysis of time course of CCR5 in ipsilateral brain of ICH+LPS group.

|  | | CCR5 | | | F(df1, df2)=  value | *P* |
| --- | --- | --- | --- | --- | --- | --- |
|  |  | 1 d | 3 d | 7 d |  |  |
| Mean | | 1 | 1.515 | 0.5943 |  |  |
| Std. Deviation | | 0 | 0.1185 | 0.1737 |  |  |
| Normality : Shapiro-Wilk normality test | W |  | 0.8765 | 0.8964 |  |  |
|  | *P* |  | 0.2535 | 0.3532 |  |  |
| Homogeneity of variances test : Levene Statistic | |  |  |  | F(2, 15)=3.986 | 0.0409 |

| CCR5 | One way ANOVA | Tukey's multiple comparisons test  *P* value |
| --- | --- | --- |
| 1 d vs. 3 d |  | <0.0001 |
| 1 d vs. 7 d |  | 0.0001 |
| 3 d vs. 7 d |  | <0.0001 |
| F | 52.46 |  |
| *P* | <0.0001 |  |

**Fig. 6** (D, E) Quantitative analysis of time course of CCL5 in ipsilateral brain and serum of ICH+LPS group.

| Cerebrum hemisphere | | CCL5 | | | F(df1, df2)=  value | *P* |
| --- | --- | --- | --- | --- | --- | --- |
|  |  | 1 d | 3 d | 7 d |  |  |
| Mean (ng/ml) | | 22.44 | 34.94 | 16.33 |  |  |
| Std. Deviation | | 1.437 | 2.663 | 1.358 |  |  |
| Normality : Shapiro-Wilk normality test | W | 0.936 | 0.9394 | 0.9213 |  |  |
|  | *P* | 0.6268 | 0.6546 | 0.5145 |  |  |
| Homogeneity of variances test : Levene Statistic | |  |  |  | F(2, 15)=3.373 | 0.0617 |
| Serum | | CCL5 | | | F(df1, df2)=  value | *P* |
|  |  | 1 d | 3 d | 7 d |  |  |
| Mean (pg/ml) | | 82.95 | 174.6 | 60.1 |  |  |
| Std. Deviation | | 5.896 | 9.01 | 6.773 |  |  |
| Normality : Shapiro-Wilk normality test | W | 0.9238 | 0.956 | 0.9325 |  |  |
|  | *P* | 0.5329 | 0.7881 | 0.5999 |  |  |
| Homogeneity of variances test : Levene Statistic | |  |  |  | F(2, 15)=0.2773 | 0.7616 |

| CCL5 in Cerebrum hemisphere | One way ANOVA | Tukey's multiple comparisons test  *P* value |
| --- | --- | --- |
| 1 d vs. 3 d |  | <0.0001 |
| 1 d vs. 7 d |  | 0.0002 |
| 3 d vs. 7 d |  | <0.0001 |
| F | 147.3 |  |
| *P* | <0.0001 |  |
| CCL5 in Serum | One way ANOVA | Tukey's multiple comparisons test  *P* value |
| 1 d vs. 3 d |  | <0.0001 |
| 1 d vs. 7 d |  | 0.0002 |
| 3 d vs. 7 d |  | <0.0001 |
| F | 408.5 |  |
| *P* | <0.0001 |  |

**Fig. 6** (F, G) Quantitative analysis of CCL5 in ipsilateral brain and serum of sham, LPS, LPS+Sham, ICH, and ICH+LPS groups on 3 days after ICH.

| Cerebrum hemisphere | | CCL5 | | | | | F(df1, df2)=  value | *P* |
| --- | --- | --- | --- | --- | --- | --- | --- | --- |
|  |  | Sham | LPS | LPS+Sham | ICH | ICH+LPS |  |  |
| Mean | | 9.441 | 13.09 | 13.21 | 23.34 | 34.94 |  |  |
| Std. Deviation | | 1.373 | 2.274 | 1.639 | 3.199 | 2.663 |  |  |
| Normality : Shapiro-Wilk normality test | W | 0.9934 | 0.9162 | 0.9895 | 0.9584 | 0.9394 |  |  |
|  | *P* | 0.9958 | 0.4782 | 0.9880 | 0.8074 | 0.6546 |  |  |
| Homogeneity of variances test : Levene Statistic | |  |  |  |  |  | F(4, 25)=2.075 | 0.1143 |
| Serum | | CCL5 | | | | | F(df1, df2)=  value | *P* |
|  |  | Sham | LPS | LPS+Sham | ICH | ICH+LPS |  |  |
| Mean | | 50.29 | 104.5 | 106.4 | 86.51 | 174.6 |  |  |
| Std. Deviation | | 6.822 | 5.036 | 5.286 | 8.237 | 9.01 |  |  |
| Normality : Shapiro-Wilk normality test | W | 0.9696 | 0.9836 | 0.9484 | 0.9255 | 0.956 |  |  |
|  | *P* | 0.8898 | 0.9678 | 0.7276 | 0.5457 | 0.7881 |  |  |
| Homogeneity of variances test : Levene Statistic | |  |  |  |  |  | F(4, 25)=0.353 | 0.8395 |

| CCL5 in Cerebrum hemisphere | One way ANOVA | Tukey's multiple comparisons test  *P* value |
| --- | --- | --- |
| Sham vs. LPS 3d |  | 0.0800 |
| Sham vs. LPS+Sham 3d |  | 0.0663 |
| Sham vs. ICH 3d |  | <0.0001 |
| Sham vs. ICH+LPS 3d |  | <0.0001 |
| LPS 3d vs. LPS+Sham 3d |  | >0.9999 |
| LPS 3d vs. ICH 3d |  | <0.0001 |
| LPS 3d vs. ICH+LPS 3d |  | <0.0001 |
| LPS+Sham 3d vs. ICH 3d |  | <0.0001 |
| LPS+Sham 3d vs. ICH+LPS 3d |  | <0.0001 |
| ICH 3d vs. ICH+LPS 3d |  | <0.0001 |
| F | 119.9 |  |
| *P* | <0.0001 |  |
| CCL5 in Serum | One way ANOVA | Tukey's multiple comparisons test  *P* value |
| Sham vs. LPS 3d |  | <0.0001 |
| Sham vs. LPS+Sham 3d |  | <0.0001 |
| Sham vs. ICH 3d |  | <0.0001 |
| Sham vs. ICH+LPS 3d |  | <0.0001 |
| LPS 3d vs. LPS+Sham 3d |  | 0.9907 |
| LPS 3d vs. ICH 3d |  | 0.0014 |
| LPS 3d vs. ICH+LPS 3d |  | <0.0001 |
| LPS+Sham 3d vs. ICH 3d |  | 0.0005 |
| LPS+Sham 3d vs. ICH+LPS 3d |  | <0.0001 |
| ICH 3d vs. ICH+LPS 3d |  | <0.0001 |
| F | 246.6 |  |
| *P* | <0.0001 |  |

**Fig. 7 Intensive peripheral inflammation upregulated CCR5/JAK2/STAT3/MMP9 signal pathway on 3 days after ICH.** (A-E) Western blot images and quantitative analysis of CCR5, p-JAK2, p-STAT3, and MMP9 in ipsilateral brain of sham, LPS, LPS+Sham, ICH, and ICH+LPS groups on 3 days after ICH. Data were expressed as mean±SD. ^#^ vs sham group, *P*<0.05; *^a^* vs LPS group, *P*<0.05; *^b^* vs LPS+Sham group, *P*<0.05; *^&^* vs ICH group, *P*<0.05; ns: no significance.

**Fig. 7** (A-E) Western blot images and quantitative analysis of CCR5, p-JAK2, p-STAT3, and MMP9 in ipsilateral brain of sham, LPS, LPS+Sham, ICH, and ICH+LPS groups on 3 days after ICH.

| CCR5 | | 3 day | | | | | F(df1, df2)=  value | *P* |
| --- | --- | --- | --- | --- | --- | --- | --- | --- |
|  |  | Sham | LPS | LPS+Sham | ICH | ICH+LPS |  |  |
| Mean | | 0.5 | 0.9331 | 1.023 | 1.218 | 1.654 |  |  |
| Std. Deviation | | 0 | 0.08908 | 0.1583 | 0.1049 | 0.1734 |  |  |
| Normality : Shapiro-Wilk normality test | W |  | 0.8413 | 0.9001 | 0.8655 | 0.9006 |  |  |
|  | *P* |  | 0.1338 | 0.3748 | 0.2090 | 0.3774 |  |  |
| Homogeneity of variances test : Levene Statistic | |  |  |  |  |  | F(4, 25)=3.365 | 0.0247 |
| p-JAK2 | | 3 days | | | | | F(df1, df2)=  value | *P* |
|  |  | Sham | LPS | LPS+Sham | ICH | ICH+LPS |  |  |
| Mean | | 0.5 | 1.031 | 1.036 | 1.353 | 1.78 |  |  |
| Std. Deviation | | 0 | 0.2705 | 0.2712 | 0.2478 | 0.2156 |  |  |
| Normality : Shapiro-Wilk normality test | W |  | 0.8218 | 0.8729 | 0.6705 | 0.9243 |  |  |
|  | *P* |  | 0.0914 | 0.2382 | 0.0030 | 0.5369 |  |  |
| Homogeneity of variances test : Levene Statistic | |  |  |  |  |  | F(4, 25)=1.598 | 0.2060 |
| p-STAT3 | | 3 day | | | | | F(df1, df2)=  value | *P* |
|  |  | Sham | LPS | LPS+Sham | ICH | ICH+LPS |  |  |
| Mean | | 0.5 | 0.8245 | 0.8305 | 1.096 | 1.396 |  |  |
| Std. Deviation | | 0 | 0.0828 | 0.1202 | 0.1392 | 0.1602 |  |  |
| Normality : Shapiro-Wilk normality test | W |  | 0.8555 | 0.9771 | 0.8354 | 0.9561 |  |  |
|  | *P* |  | 0.1742 | 0.9361 | 0.1194 | 0.7891 |  |  |
| Homogeneity of variances test : Levene Statistic | |  |  |  |  |  | F(4, 25)=4.106 | 0.0108 |
| MMP9 | | 3 days | | | | | F(df1, df2)=  value | *P* |
|  |  | Sham | LPS | LPS+Sham | ICH | ICH+LPS |  |  |
| Mean | | 0.5 | 1.334 | 1.347 | 1.909 | 2.444 |  |  |
| Std. Deviation | | 0 | 0.1995 | 0.1829 | 0.1583 | 0.1562 |  |  |
| Normality : Shapiro-Wilk normality test | W |  | 0.9854 | 0.84 | 0.8712 | 0.9684 |  |  |
|  | *P* |  | 0.9750 | 0.1303 | 0.2312 | 0.8817 |  |  |
| Homogeneity of variances test : Levene Statistic | |  |  |  |  |  | F(4, 25)=2.593 | 0.0609 |

| CCR5 | One way ANOVA | Tukey's multiple comparisons test  *P* value |
| --- | --- | --- |
| Sham vs. LPS 3d |  | <0.0001 |
| Sham vs. LPS+Sham 3d |  | <0.0001 |
| Sham vs. ICH 3d |  | <0.0001 |
| Sham vs. ICH+LPS 3d |  | <0.0001 |
| LPS 3d vs. LPS+Sham 3d |  | 0.7103 |
| LPS 3d vs. ICH 3d |  | 0.0036 |
| LPS 3d vs. ICH+LPS 3d |  | <0.0001 |
| LPS+Sham 3d vs. ICH 3d |  | 0.0700 |
| LPS+Sham 3d vs. ICH+LPS 3d |  | <0.0001 |
| ICH 3d vs. ICH+LPS 3d |  | <0.0001 |
| F | 71.77 |  |
| *P* | <0.0001 |  |
| p-JAK2 | One way ANOVA | Tukey's multiple comparisons test  *P* value |
| Sham vs. LPS 3d |  | 0.0034 |
| Sham vs. LPS+Sham 3d |  | 0.0031 |
| Sham vs. ICH 3d |  | <0.0001 |
| Sham vs. ICH+LPS 3d |  | <0.0001 |
| LPS 3d vs. LPS+Sham 3d |  | >0.9999 |
| LPS 3d vs. ICH 3d |  | 0.1299 |
| LPS 3d vs. ICH+LPS 3d |  | <0.0001 |
| LPS+Sham 3d vs. ICH 3d |  | 0.1412 |
| LPS+Sham 3d vs. ICH+LPS 3d |  | <0.0001 |
| ICH 3d vs. ICH+LPS 3d |  | 0.0231 |
| F | 26.14 |  |
| *P* | <0.0001 |  |
| p-STAT3 | One way ANOVA | Tukey's multiple comparisons test  *P* value |
| Sham vs. LPS 3d |  | <0.0001 |
| Sham vs. LPS+Sham 3d |  | <0.0001 |
| Sham vs. ICH 3d |  | <0.0001 |
| Sham vs. ICH+LPS 3d |  | <0.0001 |
| LPS 3d vs. LPS+Sham 3d |  | 0.9999 |
| LPS 3d vs. ICH 3d |  | <0.0001 |
| LPS 3d vs. ICH+LPS 3d |  | <0.0001 |
| LPS+Sham 3d vs. ICH 3d |  | <0.0001 |
| LPS+Sham 3d vs. ICH+LPS 3d |  | <0.0001 |
| ICH 3d vs. ICH+LPS 3d |  | <0.0001 |
| F | 129 |  |
| *P* | <0.0001 |  |
| MMP9 | One way ANOVA | Tukey's multiple comparisons test  *P* value |
| Sham vs. LPS 3d |  | 0.0005 |
| Sham vs. LPS+Sham 3d |  | 0.0004 |
| Sham vs. ICH 3d |  | <0.0001 |
| Sham vs. ICH+LPS 3d |  | <0.0001 |
| LPS 3d vs. LPS+Sham 3d |  | >0.9999 |
| LPS 3d vs. ICH 3d |  | 0.0034 |
| LPS 3d vs. ICH+LPS 3d |  | <0.0001 |
| LPS+Sham 3d vs. ICH 3d |  | 0.0043 |
| LPS+Sham 3d vs. ICH+LPS 3d |  | <0.0001 |
| ICH 3d vs. ICH+LPS 3d |  | 0.0011 |
| F | 50.97 |  |
| *P* | <0.0001 |  |

**Fig. 8 Administration of specific CCR5 inhibitor Maraviroc (MVC) alleviated the cerebral damages via JAK2/STAT3/MMP9 signaling pathway on 3 days after ICH with potentiated peripheral inflammation.** (A) Evans blue fluorescence images (Scale bar = 50 μm) and open field test (OFT) route track images; According to the preset parameters, the mouse route track is presented as three colors consisted of black (low speed), green (middle speed), and red (high speed). (B-E) Western blot images and quantitative analysis of p-JAK2, p-STAT3, and MMP9 in ipsilateral brain of sham, ICH, ICH+LPS, ICH+LPS+Vehicle, and ICH+LPS+MVC groups on 3 days after ICH. (F) Quantitative analysis of Evans blue extravasation. (G, H) Average velocity and BMS scores statistical analysis. Data were expressed as mean±SD. ^#^ vs sham group, *P*<0.05; *^c^* vs ICH+LPS group, *P*<0.05; *^d^* vs ICH+LPS+Vehicle group, *P*<0.05; *^&^* vs ICH group, *P*<0.05; ns: no significance.

**Fig. 8** (B-E) Western blot images and quantitative analysis of p-JAK2, p-STAT3, and MMP9 in ipsilateral brain of sham, ICH, ICH+LPS, ICH+LPS+Vehicle, and ICH+LPS+MVC groups on 3 days after ICH.

| p-JAK2 | | 3 days | | | | | F(df1, df2)=  value | *P* |
| --- | --- | --- | --- | --- | --- | --- | --- | --- |
|  |  | Sham | ICH | ICH+LPS | ICH+LPS+Vehicle | ICH+LPS+MVC |  |  |
| Mean | | 0.5 | 0.8547 | 1.241 | 1.254 | 0.7032 |  |  |
| Std. Deviation | | 0 | 0.09931 | 0.1926 | 0.1766 | 0.1196 |  |  |
| Normality : Shapiro-Wilk normality test | W |  | 0.9414 | 0.8731 | 0.9161 | 0.8697 |  |  |
|  | *P* |  | 0.6707 | 0.2389 | 0.4774 | 0.2252 |  |  |
| Homogeneity of variances test : Levene Statistic | |  |  |  |  |  | F(4, 25)=1.805 | 0.1594 |
| p-STAT3 | | 3 day | | | | | F(df1, df2)=  value | *P* |
|  |  | Sham | ICH | ICH+LPS | ICH+LPS+Vehicle | ICH+LPS+MVC |  |  |
| Mean | | 0.5 | 0.9013 | 1.237 | 1.242 | 0.8809 |  |  |
| Std. Deviation | | 0 | 0.1228 | 0.1734 | 0.1857 | 0.1312 |  |  |
| Normality : Shapiro-Wilk normality test | W |  | 0.8022 | 0.827 | 0.8649 | 0.9194 |  |  |
|  | *P* |  | 0.0616 | 0.1014 | 0.2068 | 0.5013 |  |  |
| Homogeneity of variances test : Levene Statistic | |  |  |  |  |  | F(4, 25)=1.744 | 0.1720 |
| MMP9 | | 3 days | | | | | F(df1, df2)=  value | *P* |
|  |  | Sham | ICH | ICH+LPS | ICH+LPS+Vehicle | ICH+LPS+MVC |  |  |
| Mean | | 0.5 | 0.7432 | 0.9896 | 0.9928 | 0.7143 |  |  |
| Std. Deviation | | 0 | 0.1171 | 0.2029 | 0.1732 | 0.1156 |  |  |
| Normality : Shapiro-Wilk normality test | W |  | 0.9254 | 0.9102 | 0.9147 | 0.8401 |  |  |
|  | *P* |  | 0.5452 | 0.4377 | 0.4680 | 0.1306 |  |  |
| Homogeneity of variances test : Levene Statistic | |  |  |  |  |  | F(4, 25)=2.353 | 0.0814 |

| p-JAK2 | One way ANOVA | Tukey's multiple comparisons test  *P* value |
| --- | --- | --- |
| Sham vs. ICH |  | 0.0011 |
| Sham vs. ICH+LPS |  | <0.0001 |
| Sham vs. ICH+LPS+Vehicle |  | <0.0001 |
| Sham vs. ICH+LPS+MVC |  | 0.1033 |
| ICH vs. ICH+LPS |  | 0.0004 |
| ICH vs. ICH+LPS+Vehicle |  | 0.0003 |
| ICH vs. ICH+LPS+MVC |  | 0.3279 |
| ICH+LPS vs. ICH+LPS+Vehicle |  | 0.9998 |
| ICH+LPS vs. ICH+LPS+MVC |  | <0.0001 |
| ICH+LPS+Vehicle vs. ICH+LPS+MVC |  | <0.0001 |
| F | 35.88 |  |
| *P* | <0.0001 |  |
| p-STAT3 | One way ANOVA | Tukey's multiple comparisons test  *P* value |
| Sham vs. ICH |  | 0.0003 |
| Sham vs. ICH+LPS |  | <0.0001 |
| Sham vs. ICH+LPS+Vehicle |  | <0.0001 |
| Sham vs. ICH+LPS+MVC |  | 0.0006 |
| ICH vs. ICH+LPS |  | 0.0027 |
| ICH vs. ICH+LPS+Vehicle |  | 0.0023 |
| ICH vs. ICH+LPS+MVC |  | 0.9990 |
| ICH+LPS vs. ICH+LPS+Vehicle |  | >0.9999 |
| ICH+LPS vs. ICH+LPS+MVC |  | 0.0014 |
| ICH+LPS+Vehicle vs. ICH+LPS+MVC |  | 0.0012 |
| F | 29.21 |  |
| *P* | <0.0001 |  |
| MMP9 | One way ANOVA | Tukey's multiple comparisons test  *P* value |
| Sham vs. ICH |  | 0.0431 |
| Sham vs. ICH+LPS |  | <0.0001 |
| Sham vs. ICH+LPS+Vehicle |  | <0.0001 |
| Sham vs. ICH+LPS+MVC |  | 0.0915 |
| ICH vs. ICH+LPS |  | 0.0395 |
| ICH vs. ICH+LPS+Vehicle |  | 0.0361 |
| ICH vs. ICH+LPS+MVC |  | 0.9963 |
| ICH+LPS vs. ICH+LPS+Vehicle |  | >0.9999 |
| ICH+LPS vs. ICH+LPS+MVC |  | 0.0175 |
| ICH+LPS+Vehicle vs. ICH+LPS+MVC |  | 0.0159 |
| F | 13.21 |  |
| *P* | <0.0001 |  |

**Fig. 8** (G, H) Average velocity and BMS scores statistical analysis in mice of sham, ICH, ICH+LPS, ICH+LPS+Vehicle, and ICH+LPS+MVC groups on 3 days after ICH.

| Average velocity | | 3 days | | | | | F(df1, df2)=  value | *P* |
| --- | --- | --- | --- | --- | --- | --- | --- | --- |
|  |  | Sham | ICH | ICH+LPS | ICH+LPS+Vehicle | ICH+LPS+MVC |  |  |
| Mean (cm/s) | | 6.911 | 2.499 | 1.089 | 1.09 | 2.103 |  |  |
| Std. Deviation | | 0.4748 | 0.3676 | 0.1884 | 0.1414 | 0.2454 |  |  |
| Normality : Shapiro-Wilk normality test | W | 0.9553 | 0.9634 | 0.9539 | 0.9039 | 0.9702 |  |  |
|  | *P* | 0.7832 | 0.8452 | 0.7714 | 0.3977 | 0.8939 |  |  |
| Homogeneity of variances test : Levene Statistic | |  |  |  |  |  | F(4, 25)=3.477 | 0.0217 |

| BMS Scores | 3 day | | | | |
| --- | --- | --- | --- | --- | --- |
|  | Sham | ICH | ICH+LPS | ICH+LPS+Vehicle | ICH+LPS+MVC |
| Median | 9 | 5 | 3 | 3 | 5 |
| 25% Percentile | 9 | 4 | 2 | 2 | 4 |
| 75% Percentile | 9 | 5 | 3.25 | 4.25 | 5.25 |

| Average velocity | One way ANOVA | Tukey's multiple comparisons test  *P* value |
| --- | --- | --- |
| Sham vs. ICH |  | <0.0001 |
| Sham vs. ICH+LPS |  | <0.0001 |
| Sham vs. ICH+LPS+Vehicle |  | <0.0001 |
| Sham vs. ICH+LPS+MVC |  | <0.0001 |
| ICH vs. ICH+LPS |  | <0.0001 |
| ICH vs. ICH+LPS+Vehicle |  | <0.0001 |
| ICH vs. ICH+LPS+MVC |  | 0.2036 |
| ICH+LPS vs. ICH+LPS+Vehicle |  | >0.9999 |
| ICH+LPS vs. ICH+LPS+MVC |  | <0.0001 |
| ICH+LPS+Vehicle vs. ICH+LPS+MVC |  | <0.0001 |
| F | 367 |  |
| *P* | <0.0001 |  |
| BMS Scores | Kruskal-Wallis test | Dunn's multiple comparisons test  *P* value |
| ICH+LPS+Vehicle vs. ICH+LPS |  | >0.9999 |
| ICH+LPS+Vehicle vs. ICH+LPS+MVC |  | 0.0424 |
| K | 8.827 |  |
| *P* | 0.0067 |  |

**Fig. 8** (F) Quantitative analysis of Evans blue extravasation in mice of sham, ICH, ICH+LPS, ICH+LPS+Vehicle, and ICH+LPS+MVC groups on 3 days after ICH.

| Evans blue extravasation | | 3 days | | | | | F(df1, df2)=  value | *P* |
| --- | --- | --- | --- | --- | --- | --- | --- | --- |
|  |  | Sham | ICH | ICH+LPS | ICH+LPS+Vehicle | ICH+LPS+MVC |  |  |
| Mean (ng/mg) | | 11.23 | 21.74 | 29.12 | 30.56 | 21.21 |  |  |
| Std. Deviation | | 0.6497 | 1.983 | 2.893 | 2.574 | 3.053 |  |  |
| Normality : Shapiro-Wilk normality test | W | 0.9347 | 0.9709 | 0.8625 | 0.9707 | 0.9431 |  |  |
|  | *P* | 0.6167 | 0.8982 | 0.1979 | 0.8971 | 0.6844 |  |  |
| Homogeneity of variances test : Levene Statistic | |  |  |  |  |  | F(4, 25)=2.491 | 0.0689 |

| Evans blue extravasation | One way ANOVA | Tukey's multiple comparisons test  *P* value |
| --- | --- | --- |
| Sham vs. ICH |  | <0.0001 |
| Sham vs. ICH+LPS |  | <0.0001 |
| Sham vs. ICH+LPS+Vehicle |  | <0.0001 |
| Sham vs. ICH+LPS+MVC |  | <0.0001 |
| ICH vs. ICH+LPS |  | 0.0001 |
| ICH vs. ICH+LPS+Vehicle |  | <0.0001 |
| ICH vs. ICH+LPS+MVC |  | 0.9951 |
| ICH+LPS vs. ICH+LPS+Vehicle |  | 0.8313 |
| ICH+LPS vs. ICH+LPS+MVC |  | <0.0001 |
| ICH+LPS+Vehicle vs. ICH+LPS+MVC |  | <0.0001 |
| F | 62.19 |  |
| *P* | <0.0001 |  |

**Fig. 9 Administration of specific CCR5 agonist rCCL5 accentuated the cerebral damages via JAK2/STAT3/MMP9 signaling pathway on 3 days after ICH with intensive peripheral inflammation.** (A) Evans blue fluorescence images (Scale bar = 50 μm) and open field test (OFT) route track images; According to the preset parameters, the mouse route track is presented as three colors consisted of black (low speed), green (middle speed), and red (high speed). (B-E) Western blot images and quantitative analysis of p-JAK2, p-STAT3, and MMP9 in ipsilateral brain of sham, ICH, ICH+LPS, ICH+LPS+Vehicle, and ICH+LPS+ rCCL5 groups on 3 days after ICH. (F) Quantitative analysis of Evans blue extravasation. (G, H) Average velocity and BMS scores statistical analysis. Data were expressed as mean±SD. ^#^ vs sham group, *P*<0.05; *^c^* vs ICH+LPS group, *P*<0.05; *^d^* vs ICH+LPS+Vehicle group, *P*<0.05; *^&^* vs ICH group, *P*<0.05; ns: no significance.

**Fig. 9** (B-E) Western blot images and quantitative analysis of p-JAK2, p-STAT3, and MMP9 in ipsilateral brain of sham, ICH, ICH+LPS, ICH+LPS+Vehicle, and ICH+LPS+ rCCL5 groups on 3 days after ICH.

| p-JAK2 | | 3 days | | | | | F(df1, df2)=  value | *P* |
| --- | --- | --- | --- | --- | --- | --- | --- | --- |
|  |  | Sham | ICH | ICH+LPS | ICH+LPS+Vehicle | ICH+LPS+rCCL5 |  |  |
| Mean | | 0.5 | 0.8516 | 1.088 | 1.097 | 1.577 |  |  |
| Std. Deviation | | 0 | 0.06269 | 0.0485 | 0.1091 | 0.2095 |  |  |
| Normality : Shapiro-Wilk normality test | W |  | 0.9634 | 0.9818 | 0.9824 | 0.9362 |  |  |
|  | *P* |  | 0.8453 | 0.9600 | 0.9629 | 0.6287 |  |  |
| Homogeneity of variances test : Levene Statistic | |  |  |  |  |  | F(4, 25)=5.095 | 0.0038 |
| p-STAT3 | | 3 day | | | | | F(df1, df2)=  value | *P* |
|  |  | Sham | ICH | ICH+LPS | ICH+LPS+Vehicle | ICH+LPS+ rCCL5 |  |  |
| Mean | | 0.5 | 0.8079 | 1.148 | 1.187 | 1.718 |  |  |
| Std. Deviation | | 0 | 0.1007 | 0.06224 | 0.1073 | 0.1709 |  |  |
| Normality : Shapiro-Wilk normality test | W |  | 0.8628 | 0.9101 | 0.7832 | 0.9119 |  |  |
|  | *P* |  | 0.1989 | 0.4374 | 0.0413 | 0.4490 |  |  |
| Homogeneity of variances test : Levene Statistic | |  |  |  |  |  | F(4, 25)=2.252 | 0.0920 |
| MMP9 | | 3 days | | | | | F(df1, df2)=  value | *P* |
|  |  | Sham | ICH | ICH+LPS | ICH+LPS+Vehicle | ICH+LPS+ rCCL5 |  |  |
| Mean | | 0.5 | 0.8454 | 1.182 | 1.141 | 1.735 |  |  |
| Std. Deviation | | 0 | 0.1541 | 0.1074 | 0.08424 | 0.1363 |  |  |
| Normality : Shapiro-Wilk normality test | W |  | 0.9062 | 0.7519 | 0.9272 | 0.9597 |  |  |
|  | *P* |  | 0.4117 | 0.0208 | 0.5584 | 0.8175 |  |  |
| Homogeneity of variances test : Levene Statistic | |  |  |  |  |  | F(4, 25)=3.682 | 0.0172 |

| p-JAK2 | One way ANOVA | Tukey's multiple comparisons test  *P* value |
| --- | --- | --- |
| Sham vs. ICH |  | 0.0001 |
| Sham vs. ICH+LPS |  | <0.0001 |
| Sham vs. ICH+LPS+Vehicle |  | <0.0001 |
| Sham vs. ICH+LPS+rCCL5 |  | <0.0001 |
| ICH vs. ICH+LPS |  | 0.0090 |
| ICH vs. ICH+LPS+Vehicle |  | 0.0064 |
| ICH vs. ICH+LPS+rCCL5 |  | <0.0001 |
| ICH+LPS vs. ICH+LPS+Vehicle |  | >0.9999 |
| ICH+LPS vs. ICH+LPS+rCCL5 |  | <0.0001 |
| ICH+LPS+Vehicle vs. ICH+LPS+rCCL5 |  | <0.0001 |
| F | 74.87 |  |
| *P* | <0.0001 |  |
| p-STAT3 | One way ANOVA | Tukey's multiple comparisons test  *P* value |
| Sham vs. ICH |  | 0.0003 |
| Sham vs. ICH+LPS |  | <0.0001 |
| Sham vs. ICH+LPS+Vehicle |  | <0.0001 |
| Sham vs. ICH+LPS+rCCL5 |  | <0.0001 |
| ICH vs. ICH+LPS |  | <0.0001 |
| ICH vs. ICH+LPS+Vehicle |  | <0.0001 |
| ICH vs. ICH+LPS+rCCL5 |  | <0.0001 |
| ICH+LPS vs. ICH+LPS+Vehicle |  | 0.9669 |
| ICH+LPS vs. ICH+LPS+rCCL5 |  | <0.0001 |
| ICH+LPS+Vehicle vs. ICH+LPS+rCCL5 |  | <0.0001 |
| F | 114.1 |  |
| *P* | <0.0001 |  |
| MMP9 | One way ANOVA | Tukey's multiple comparisons test  *P* value |
| Sham vs. ICH |  | 0.0001 |
| Sham vs. ICH+LPS |  | <0.0001 |
| Sham vs. ICH+LPS+Vehicle |  | <0.0001 |
| Sham vs. ICH+LPS+rCCL5 |  | <0.0001 |
| ICH vs. ICH+LPS |  | 0.0002 |
| ICH vs. ICH+LPS+Vehicle |  | 0.0008 |
| ICH vs. ICH+LPS+rCCL5 |  | <0.0001 |
| ICH+LPS vs. ICH+LPS+Vehicle |  | 0.9660 |
| ICH+LPS vs. ICH+LPS+rCCL5 |  | <0.0001 |
| ICH+LPS+Vehicle vs. ICH+LPS+rCCL5 |  | <0.0001 |
| F | 102.7 |  |
| *P* | <0.0001 |  |

**Fig. 9** (F) Quantitative analysis of Evans blue extravasation in mice of sham, ICH, ICH+LPS, ICH+LPS+Vehicle, and ICH+LPS+ rCCL5 groups on 3 days after ICH.

| Evans blue extravasation | | 3 days | | | | | F(df1, df2)=  value | *P* |
| --- | --- | --- | --- | --- | --- | --- | --- | --- |
|  |  | Sham | ICH | ICH+LPS | ICH+LPS+Vehicle | ICH+LPS+ rCCL5 |  |  |
| Mean (ng/mg) | | 11.23 | 21.74 | 29.12 | 30.56 | 37.22 |  |  |
| Std. Deviation | | 0.6497 | 1.983 | 2.893 | 2.574 | 2.038 |  |  |
| Normality : Shapiro-Wilk normality test | W | 0.9347 | 0.9709 | 0.8625 | 0.9707 | 0.8923 |  |  |
|  | *P* | 0.6167 | 0.8982 | 0.1979 | 0.8971 | 0.3306 |  |  |
| Homogeneity of variances test : Levene Statistic | |  |  |  |  |  | F(4, 25)=2.702 | 0.0535 |

| Evans blue extravasation | One way ANOVA | Tukey's multiple comparisons test  *P* value |
| --- | --- | --- |
| Sham vs. ICH |  | <0.0001 |
| Sham vs. ICH+LPS |  | <0.0001 |
| Sham vs. ICH+LPS+Vehicle |  | <0.0001 |
| Sham vs. ICH+LPS+rCCL5 |  | <0.0001 |
| ICH vs. ICH+LPS |  | <0.0001 |
| ICH vs. ICH+LPS+Vehicle |  | <0.0001 |
| ICH vs. ICH+LPS+rCCL5 |  | <0.0001 |
| ICH+LPS vs. ICH+LPS+Vehicle |  | 0.7756 |
| ICH+LPS vs. ICH+LPS+rCCL5 |  | <0.0001 |
| ICH+LPS+Vehicle vs. ICH+LPS+rCCL5 |  | 0.0001 |
| F | 125.4 |  |
| *P* | <0.0001 |  |

**Fig. 9** (G, H) Average velocity and BMS scores statistical analysis in mice of sham, ICH, ICH+LPS, ICH+LPS+Vehicle, and ICH+LPS+ rCCL5 groups on 3 days after ICH.

| Average velocity | | 3 days | | | | | F(df1, df2)=  value | *P* |
| --- | --- | --- | --- | --- | --- | --- | --- | --- |
|  |  | Sham | ICH | ICH+LPS | ICH+LPS+Vehicle | ICH+LPS+ rCCL5 |  |  |
| Mean (cm/s) | | 6.911 | 2.499 | 1.089 | 1.09 | 0.1843 |  |  |
| Std. Deviation | | 0.4748 | 0.3676 | 0.1884 | 0.1414 | 0.05977 |  |  |
| Normality : Shapiro-Wilk normality test | W | 0.9553 | 0.9634 | 0.9539 | 0.9039 | 0.9554 |  |  |
|  | *P* | 0.7832 | 0.8452 | 0.7714 | 0.3977 | 0.7835 |  |  |
| Homogeneity of variances test : Levene Statistic | |  |  |  |  |  | F(4, 25)=6.359 | 0.0011 |

| BMS Scores | 3 day | | | | |
| --- | --- | --- | --- | --- | --- |
|  | Sham | ICH | ICH+LPS | ICH+LPS+Vehicle | ICH+LPS+ rCCL5 |
| Median | 9 | 5 | 3 | 3 | 2 |
| 25% Percentile | 9 | 4 | 2 | 3 | 2 |
| 75% Percentile | 9 | 5 | 3.25 | 4.25 | 2.25 |

| Average velocity | One way ANOVA | Tukey's multiple comparisons test  *P* value |
| --- | --- | --- |
| Sham vs. ICH |  | <0.0001 |
| Sham vs. ICH+LPS |  | <0.0001 |
| Sham vs. ICH+LPS+Vehicle |  | <0.0001 |
| Sham vs. ICH+LPS+rCCL5 |  | <0.0001 |
| ICH vs. ICH+LPS |  | <0.0001 |
| ICH vs. ICH+LPS+Vehicle |  | <0.0001 |
| ICH vs. ICH+LPS+rCCL5 |  | <0.0001 |
| ICH+LPS vs. ICH+LPS+Vehicle |  | >0.9999 |
| ICH+LPS vs. ICH+LPS+rCCL5 |  | 0.0001 |
| ICH+LPS+Vehicle vs. ICH+LPS+rCCL5 |  | 0.0001 |
| F | 512.7 |  |
| *P* | <0.0001 |  |
| BMS Scores | Kruskal-Wallis test | Dunn's multiple comparisons test  *P* value |
| ICH+LPS+Vehicle vs. ICH+LPS |  | 0.4170 |
| ICH+LPS+Vehicle vs. ICH+LPS+rCCL5 |  | 0.0083 |
| K | 8.257 |  |
| P | 0.0142 |  |

**Fig. 10 JAK2 CRISPR knockdown reversed the cerebral damages of intensive peripheral inflammation on 3 days after ICH via inhibiting JAK2/STAT3/MMP9 signal pathway.** (A) Evans blue fluorescence images (Scale bar = 50 μm) and open field test (OFT) route track images; According to the preset parameters, the mouse route track is presented as three colors consisted of black (low speed), green (middle speed), and red (high speed). (B-E) Western blot images and quantitative analysis of JAK2, p-STAT3, and MMP9 in ipsilateral brain of sham, ICH+LPS+Vehicle, ICH+LPS+rCCL5, ICH+LPS+rCCL5+Ctr CRISPR, and ICH+LPS+rCCL5+JAK2 CRISPR groups on 3 days after ICH. (F) Quantitative analysis of Evans blue extravasation. (G, H) Average velocity and BMS scores statistical analysis. Data were expressed as mean±SD. ^#^ vs sham group, *P*<0.05; *^d^* vs ICH+LPS+Vehicle group, *P*<0.05; *^e^* vs ICH+LPS+rCCL5 group, *P*<0.05; *^f^* vs ICH+LPS+rCCL5 +Ctr CRISPR group, *P*<0.05; ns: no significance. Ctr: control.

**Fig. 10** (B-E) Western blot images and quantitative analysis of JAK2, p-STAT3, and MMP9 in ipsilateral brain of sham, ICH+LPS+Vehicle, ICH+LPS+rCCL5, ICH+LPS+rCCL5+Ctr CRISPR, and ICH+LPS+rCCL5+JAK2 CRISPR groups on 3 days after ICH.

| JAK2 | | Sham | ICH+LPS | | | | F(df1, df2)=  value | *P* |
| --- | --- | --- | --- | --- | --- | --- | --- | --- |
|  |  |  | +Vehicle | +rCCL5 | +rCCL5  +Ctr CRISPR | +rCCL5  +JAK2 CRISPR |  |  |
| Mean | | 0.5 | 0.9883 | 1.354 | 1.354 | 0.9322 |  |  |
| Std. Deviation | | 0 | 0.1057 | 0.2457 | 0.2514 | 0.1256 |  |  |
| Normality : Shapiro-Wilk normality test | W |  | 0.9273 | 0.7882 | 0.8218 | 0.9777 |  |  |
|  | *P* |  | 0.5594 | 0.0459 | 0.0914 | 0.9395 |  |  |
| Homogeneity of variances test : Levene Statistic | |  |  |  |  |  | F(4, 25)=24.01 | <0.0001 |
| p-STAT3 | | Sham | ICH+LPS | | | | F(df1, df2)=  value | *P* |
|  |  |  | +Vehicle | +rCCL5 | +rCCL5  +Ctr CRISPR | +rCCL5  +JAK2 CRISPR |  |  |
| Mean | | 0.5 | 0.8947 | 1.231 | 1.238 | 0.9086 |  |  |
| Std. Deviation | | 0 | 0.1089 | 0.2012 | 0.2053 | 0.1291 |  |  |
| Normality : Shapiro-Wilk normality test | W |  | 0.9134 | 0.9022 | 0.9597 | 0.9297 |  |  |
|  | *P* |  | 0.4592 | 0.3873 | 0.8176 | 0.5776 |  |  |
| Homogeneity of variances test : Levene Statistic | |  |  |  |  |  | F(4, 25)=2.026 | 0.1214 |
| MMP9 | | Sham | ICH+LPS | | | |  |  |
|  |  |  | +Vehicle | +rCCL5 | +rCCL5  +Ctr CRISPR | +rCCL5  +JAK2 CRISPR |  |  |
| Mean | | 0.5 | 2.055 | 2.933 | 2.939 | 1.793 |  |  |
| Std. Deviation | | 0 | 0.1966 | 0.1852 | 0.2084 | 0.1847 |  |  |
| Normality : Shapiro-Wilk normality test | W |  | 0.7886 | 0.9746 | 0.8206 | 0.9189 |  |  |
|  | *P* |  | 0.0463 | 0.9219 | 0.0894 | 0.4978 |  |  |
| Homogeneity of variances test : Levene Statistic | |  |  |  |  |  | F(4, 25)=2.109 | 0.1097 |

| JAK2 | One way ANOVA | Tukey's multiple comparisons test  *P* value |
| --- | --- | --- |
| Sham vs. ICH+LPS+Vehicle |  | 0.0005 |
| Sham vs. ICH+LPS+rCCL5 |  | <0.0001 |
| Sham vs. ICH+LPS+rCCL5+Ctr CRISPR |  | <0.0001 |
| Sham vs. ICH+LPS+rCCL5+JAK2 CRISPR |  | 0.0019 |
| ICH+LPS+Vehicle vs. ICH+LPS+rCCL5 |  | 0.0097 |
| ICH+LPS+Vehicle vs. ICH+LPS+rCCL5+Ctr CRISPR |  | 0.0096 |
| ICH+LPS+Vehicle vs. ICH+LPS+rCCL5+JAK2 CRISPR |  | 0.9796 |
| ICH+LPS+rCCL5 vs. ICH+LPS+rCCL5+Ctr CRISPR |  | >0.9999 |
| ICH+LPS+rCCL5 vs. ICH+LPS+rCCL5+JAK2 CRISPR |  | 0.0024 |
| ICH+LPS+rCCL5+Ctr CRISPR vs. ICH+LPS+rCCL5+JAK2 CRISPR |  | 0.0024 |
| F | 25.03 |  |
| *P* | <0.0001 |  |
| p-STAT3 | One way ANOVA | Tukey's multiple comparisons test  *P* value |
| Sham vs. ICH+LPS+Vehicle |  | 0.0010 |
| Sham vs. ICH+LPS+rCCL5 |  | <0.0001 |
| Sham vs. ICH+LPS+rCCL5+Ctr CRISPR |  | <0.0001 |
| Sham vs. ICH+LPS+rCCL5+JAK2 CRISPR |  | 0.0006 |
| ICH+LPS+Vehicle vs. ICH+LPS+rCCL5 |  | 0.0052 |
| ICH+LPS+Vehicle vs. ICH+LPS+rCCL5+Ctr CRISPR |  | 0.0042 |
| ICH+LPS+Vehicle vs. ICH+LPS+rCCL5+JAK2 CRISPR |  | 0.9998 |
| ICH+LPS+rCCL5 vs. ICH+LPS+rCCL5+Ctr CRISPR |  | >0.9999 |
| ICH+LPS+rCCL5 vs. ICH+LPS+rCCL5+JAK2 CRISPR |  | 0.0077 |
| ICH+LPS+rCCL5+Ctr CRISPR vs. ICH+LPS+rCCL5+JAK2 CRISPR |  | 0.0063 |
| F | 24.92 |  |
| *P* | <0.0001 |  |
| MMP9 | One way ANOVA | Tukey's multiple comparisons test  *P* value |
| Sham vs. ICH+LPS+Vehicle |  | <0.0001 |
| Sham vs. ICH+LPS+rCCL5 |  | <0.0001 |
| Sham vs. ICH+LPS+rCCL5+Ctr CRISPR |  | <0.0001 |
| Sham vs. ICH+LPS+rCCL5+JAK2 CRISPR |  | <0.0001 |
| ICH+LPS+Vehicle vs. ICH+LPS+rCCL5 |  | <0.0001 |
| ICH+LPS+Vehicle vs. ICH+LPS+rCCL5+Ctr CRISPR |  | <0.0001 |
| ICH+LPS+Vehicle vs. ICH+LPS+rCCL5+JAK2 CRISPR |  | 0.0967 |
| ICH+LPS+rCCL5 vs. ICH+LPS+rCCL5+Ctr CRISPR |  | >0.9999 |
| ICH+LPS+rCCL5 vs. ICH+LPS+rCCL5+JAK2 CRISPR |  | <0.0001 |
| ICH+LPS+rCCL5+Ctr CRISPR vs. ICH+LPS+rCCL5+JAK2 CRISPR |  | <0.0001 |
| F | 201.4 |  |
| *P* | <0.0001 |  |

**Fig. 10** (F) Quantitative analysis of Evans blue extravasation in the mice of sham, ICH+LPS+Vehicle, ICH+LPS+rCCL5, ICH+LPS+rCCL5+Ctr CRISPR, and ICH+LPS+rCCL5+JAK2 CRISPR groups on 3 days after ICH.

| Evans blue extravasation | | Sham | ICH+LPS | | | | F(df1, df2)=  value | *P* |
| --- | --- | --- | --- | --- | --- | --- | --- | --- |
|  |  |  | +Vehicle | +rCCL5 | +rCCL5  +Ctr CRISPR | +rCCL5  +JAK2 CRISPR |  |  |
| Mean (ng/mg) | | 11.23 | 30.56 | 37.22 | 37.13 | 25.18 |  |  |
| Std. Deviation | | 0.6477 | 2.574 | 2.038 | 1.985 | 1.575 |  |  |
| Normality : Shapiro-Wilk normality test | W | 0.9345 | 0.9706 | 0.8926 | 0.9568 | 0.8838 |  |  |
|  | *P* | 0.6152 | 0.8962 | 0.3323 | 0.7946 | 0.2869 |  |  |
| Homogeneity of variances test : Levene Statistic | |  |  |  |  |  | F(4, 25)=2.187 | 0.0997 |

| Evans blue extravasation | One way ANOVA | Tukey's multiple comparisons test  *P* value |
| --- | --- | --- |
| Sham vs. ICH+LPS+Vehicle |  | <0.0001 |
| Sham vs. ICH+LPS+rCCL5 |  | <0.0001 |
| Sham vs. ICH+LPS+rCCL5+Ctr CRISPR |  | <0.0001 |
| Sham vs. ICH+LPS+rCCL5+JAK2 CRISPR |  | <0.0001 |
| ICH+LPS+Vehicle vs. ICH+LPS+rCCL5 |  | <0.0001 |
| ICH+LPS+Vehicle vs. ICH+LPS+rCCL5+Ctr CRISPR |  | <0.0001 |
| ICH+LPS+Vehicle vs. ICH+LPS+rCCL5+JAK2 CRISPR |  | 0.0004 |
| ICH+LPS+rCCL5 vs. ICH+LPS+rCCL5+Ctr CRISPR |  | >0.9999 |
| ICH+LPS+rCCL5 vs. ICH+LPS+rCCL5+JAK2 CRISPR |  | <0.0001 |
| ICH+LPS+rCCL5+Ctr CRISPR vs. ICH+LPS+rCCL5+JAK2 CRISPR |  | <0.0001 |
| F | 197.4 |  |
| *P* | <0.0001 |  |

**Fig. 10** (G, H) Average velocity and BMS scores statistical analysis for the mice of sham, ICH+LPS+Vehicle, ICH+LPS+rCCL5, ICH+LPS+rCCL5+Ctr CRISPR, and ICH+LPS+rCCL5+JAK2 CRISPR groups on 3 days after ICH.

| Average Velocity | | Sham | ICH+LPS | | | | F(df1, df2)=  value | *P* |
| --- | --- | --- | --- | --- | --- | --- | --- | --- |
|  |  |  | +Vehicle | +rCCL5 | +rCCL5  +Ctr CRISPR | +rCCL5  +JAK2 CRISPR |  |  |
| Mean (cm/s) | | 6.911 | 1.09 | 0.1843 | 0.1932 | 1.941 |  |  |
| Std. Deviation | | 0.4748 | 0.1414 | 0.05977 | 0.01641 | 0.1715 |  |  |
| Normality : Shapiro-Wilk normality test | W | 0.9553 | 0.9039 | 0.9554 | 0.9753 | 0.9445 |  |  |
|  | *P* | 0.7832 | 0.3977 | 0.7835 | 0.9259 | 0.6953 |  |  |
| Homogeneity of variances test : Levene Statistic | |  |  |  |  |  | F(4, 25)=9.279 | <0.0001 |

| BMS Scores | Sham | ICH+LPS | | | |
| --- | --- | --- | --- | --- | --- |
|  |  | +Vehicle | +rCCL5 | +rCCL5  +Ctr CRISPR | +rCCL5  +JAK2 CRISPR |
| Median | 9 | 3 | 2 | 2.5 | 5 |
| 25% Percentile | 9 | 2 | 2 | 1.75 | 4 |
| 75% Percentile | 9 | 4.25 | 2.25 | 3 | 5.25 |

| Average Velocity | One way ANOVA | Tukey's multiple comparisons test  *P* value |
| --- | --- | --- |
| Sham vs. ICH+LPS+Vehicle |  | <0.0001 |
| Sham vs. ICH+LPS+rCCL5 |  | <0.0001 |
| Sham vs. ICH+LPS+rCCL5+Ctr CRISPR |  | <0.0001 |
| Sham vs. ICH+LPS+rCCL5+JAK2 CRISPR |  | <0.0001 |
| ICH+LPS+Vehicle vs. ICH+LPS+rCCL5 |  | <0.0001 |
| ICH+LPS+Vehicle vs. ICH+LPS+rCCL5+Ctr CRISPR |  | <0.0001 |
| ICH+LPS+Vehicle vs. ICH+LPS+rCCL5+JAK2 CRISPR |  | <0.0001 |
| ICH+LPS+rCCL5 vs. ICH+LPS+rCCL5+Ctr CRISPR |  | >0.9999 |
| ICH+LPS+rCCL5 vs. ICH+LPS+rCCL5+JAK2 CRISPR |  | <0.0001 |
| ICH+LPS+rCCL5+Ctr CRISPR vs. ICH+LPS+rCCL5+JAK2 CRISPR |  | <0.0001 |
| F | 847.3 |  |
| *P* | <0.0001 |  |
| BMS Scores | Kruskal-Wallis test | Dunn's multiple comparisons test  *P* value |
| ICH+LPS+rCCL5 vs. ICH+LPS+rCCL5+Ctr CRISPR |  | >0.9999 |
| ICH+LPS+rCCL5+Ctr CRISPR vs. ICH+LPS+rCCL5+JAK2 CRISPR |  | 0.0091 |
| K | 12.42 |  |
| *P* | 0.0001 |  |
